# hESC‐Derived Epicardial Cells Promote Repair of Infarcted Hearts in Mouse and Swine

**DOI:** 10.1002/advs.202300470

**Published:** 2023-07-28

**Authors:** Xiao‐Ling Luo, Yun Jiang, Qiang Li, Xiu‐Jian Yu, Teng Ma, Hao Cao, Min‐Xia Ke, Peng Zhang, Ji‐Liang Tan, Yan‐Shan Gong, Li Wang, Ling Gao, Huang‐Tian Yang

**Affiliations:** ^1^ CAS Key Laboratory of Tissue Microenvironment and Tumor Laboratory of Molecular Cardiology Shanghai Institute of Nutrition and Health University of Chinese Academy of Sciences (CAS) Shanghai 200031 P. R. China; ^2^ Translational Medical Center for Stem Cell Therapy, Institutes for Regenerative Medicine and Heart Failure Shanghai East Hospital Tongji University School of Medicine Shanghai 200123 China; ^3^ Department of Cardiovascular and Thoracic Surgery Shanghai East Hospital Tongji University School of Medicine Shanghai 200120 China; ^4^ State Key Laboratory of Cardiovascular Disease Fuwai Hospital National Center for Cardiovascular Diseases Chinese Academy of Medical Sciences and Peking Union Medical College Beijing 100037 China; ^5^ Institute for Stem Cell and Regeneration CAS Beijing 100101 China

**Keywords:** hESC‐derived epicardial cells, inflammation, myocardial infarction, paracrine factor, type I interferon signaling

## Abstract

Myocardial infarction (MI) causes excessive damage to the myocardium, including the epicardium. However, whether pluripotent stem cell‐derived epicardial cells (EPs) can be a therapeutic approach for infarcted hearts remains unclear. Here, the authors report that intramyocardial injection of human embryonic stem cell‐derived EPs (hEPs) at the acute phase of MI ameliorates functional worsening and scar formation in mouse hearts, concomitantly with enhanced cardiomyocyte survival, angiogenesis, and lymphangiogenesis. Mechanistically, hEPs suppress MI‐induced infiltration and cytokine‐release of inflammatory cells and promote reparative macrophage polarization. These effects are blocked by a type I interferon (IFN‐I) receptor agonist RO8191. Moreover, intelectin 1 (ITLN1), abundantly secreted by hEPs, interacts with IFN‐β and mimics the effects of hEP‐conditioned medium in suppression of IFN‐β‐stimulated responses in macrophages and promotion of reparative macrophage polarization, whereas ITLN1 downregulation in hEPs cancels beneficial effects of hEPs in anti‐inflammation, IFN‐I response inhibition, and cardiac repair. Further, similar beneficial effects of hEPs are observed in a clinically relevant porcine model of reperfused MI, with no increases in the risk of hepatic, renal, and cardiac toxicity. Collectively, this study reveals hEPs as an inflammatory modulator in promoting infarct healing via a paracrine mechanism and provides a new therapeutic approach for infarcted hearts.

## Graphical abstract

Implantation of human embryonic stem cell‐derived epicardial cells (hEPs) favors repair of infarcted murine and porcine hearts. The beneficial effects are related to hEP‐secreted ITLN1, which interacts with IFN‐β and subsequently inhibits type I interferon (IFN‐I) signaling‐mediated inflammatory responses in macrophages, resulting in promoting reparative inflammation and infarct healing. The findings reveal hEPs as a therapeutic option for infarcted hearts.

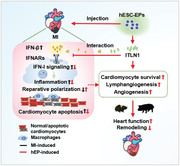

## 1 Introduction

Myocardial infarction (MI) causes irreversible loss of myocardial contractile tissue that far exceeds the limited regeneration capacity, resulting in heart failure. In an attempt to trigger endogenous repair mechanisms and/or replenish lost cardiomyocytes, cell therapies implanting human pluripotent stem cell (hPSC)‐derived cardiovascular progenitor cells (hCVPCs) or cardiomyocytes (hCMs) into infarcted hearts have been tested in preclinical models^[^ ^1^, ^2^, ^3^, ^4^, ^5^, ^6^, ^7^, ^8^ ^]^ and clinical studies.^[^ ^9^, ^10^ ^]^ As the destruction/reactivation of non‐cardiomyocytes, such as blood vessels, lymphangion, and epicardium, also occurs following MI,^[^ ^11^, ^12^ ^]^ coordinating regulation of these endogenous cells/tissues with hPSC‐based cell therapy strategies may promote restoration of cardiac function and regeneration of damaged myocardium. In support of this notion, delivery of hCVPCs^[^ ^4^, ^13^ ^]^ and endothelial cells^[^ ^14^, ^15^ ^]^ promotes the repair of infarcted hearts with the concomitant improvement in myocardial vascularization. Therefore, efforts to determine the precise therapeutic effects of non‐cardiomyocytes in the heart and to understand the underlying mechanisms may extend therapeutic options for the repair and regeneration of infarcted hearts.

Emerging evidence suggests that the MI‐activated endogenous epicardium acts as a regenerative signal reservoir via epithelial–mesenchymal transition (EMT) and secreting paracrine factors.^[^ ^16^, ^17^, ^18^, ^19^ ^]^ In addition, implantation of epicardium‐derived cells (EPDCs) isolated from human adult atrial tissues preserves left ventricular (LV) function and attenuates remodeling in infarcted mouse hearts.^[^ ^20^, ^21^ ^]^ These findings raise the possibility that adult mammalian epicardium might have therapeutic benefits for infarcted hearts, whereas challenges remain with regard to cell acquisition and expansion. Recently, human embryonic stem cell (hESC)‐derived epicardial cells (hEPs) were demonstrated to increase cardiac graft size and host vascularization when co‐transplanted with hCMs at the sub‐acute phase in a rat ischemia/reperfusion (I/R) model.^[^ ^22^ ^]^ However, it remains unclear whether the implantation of hEPs has therapeutic effects on infarcted hearts.

Acute MI induces inflammatory cascades via the recruitment of multiple inflammatory and immune cells, leading to multifaceted processes of myocardial injury and healing.^[^ ^4^, ^23^, ^24^ ^]^ Macrophages are critical responders at the acute phase of MI, which are activated and polarized into classical pro‐inflammatory (M1‐like, marked by the secretion of inflammatory cytokines and chemokines) or alternatively, reparative (M2‐like, marked by the secretion of interleukin [IL]−10, IL‐13, transforming growth factor‐β [TGF‐β], and proangiogenic factors) phenotypes.^[^ ^4^, ^25^, ^26^ ^]^ Modulation of inflammation at the acute phase of MI benefits the infarcted hearts in animal models.^[^ ^23^, ^27^, ^28^ ^]^ Favorable shifts of macrophages to the reparative phenotype exhibit therapeutic potential in the infarcted hearts.^[^ ^4^, ^29^, ^30^, ^31^ ^]^ However, it is unknown whether hEPs can regulate immune responses, especially macrophage plasticity, at the acute phase of MI.

Type I interferons (IFN‐I) are cytokines that have antiviral, antiproliferative, and immunomodulatory activities.^[^ ^32^, ^33^ ^]^ As one of the best characterized IFN‐I, IFN‐α is predominantly produced by plasmacytoid dendritic cells, while IFN‐β comes from multiple cell types, such as monocytes/macrophages, dendritic cells, and fibroblasts.^[^ ^33^, ^34^, ^35^ ^]^ IFN‐I signaling, initiated upon the binding to the IFN‐I receptors (IFNARs, composed of IFNAR1 and IFNAR2 subunits^[^ ^33^ ^]^), activates a powerful inflammatory program of IFN‐stimulated genes (ISGs), such as MX dynamin like GTPase 1 (*Mx1*), interferon induced proteins with tetratricopeptide repeats 1 (*Ifit1*), and 2′‐5′‐oligoadenylate synthetase (*Oas)*, which subsequently aggravates MI‐induced cardiac injury.^[^ ^36^ ^]^ IFN‐I responses are upregulated at the early stage of MI, and the blockade of them by IFNAR‐neutralizing antibodies improves LV function and mouse survival.^[^ ^36^ ^]^ Consistently, selective inhibitors against components of the IFN‐I pathway reduce infarct size.^[^ ^35^ ^]^ However, little is known about whether the IFN‐I ligands can be directly regulated by interacting with secreted factors and whether hEP‐secreted paracrine factors participate in this process.

In this study, we demonstrated the therapeutic effects of hEPs after intramyocardially injecting into murine and porcine MI hearts. Combined with integrated approaches, including the manipulation of IFN‐I responses and the hEP‐secreted factor, we revealed previously unknown mechanisms underlying the beneficial effects of hEPs in orchestrating IFN‐I‐mediated inflammatory responses via paracrine factors. We further identified that intelectin 1 (ITLN1), abundantly secreted from hEPs, interacted with IFN‐β, resulting in the suppression of inflammation and the promotion of cardiac repair. The novel function and mechanism of hEPs uncovered here provide preclinical insights into a new option for promoting infarct healing and favor the development of cell‐based therapeutic strategies for the treatment of ischemic heart diseases.

## 2 Results

### 2.1 Differentiation and Characterization of hEPs

**Figure 1:**
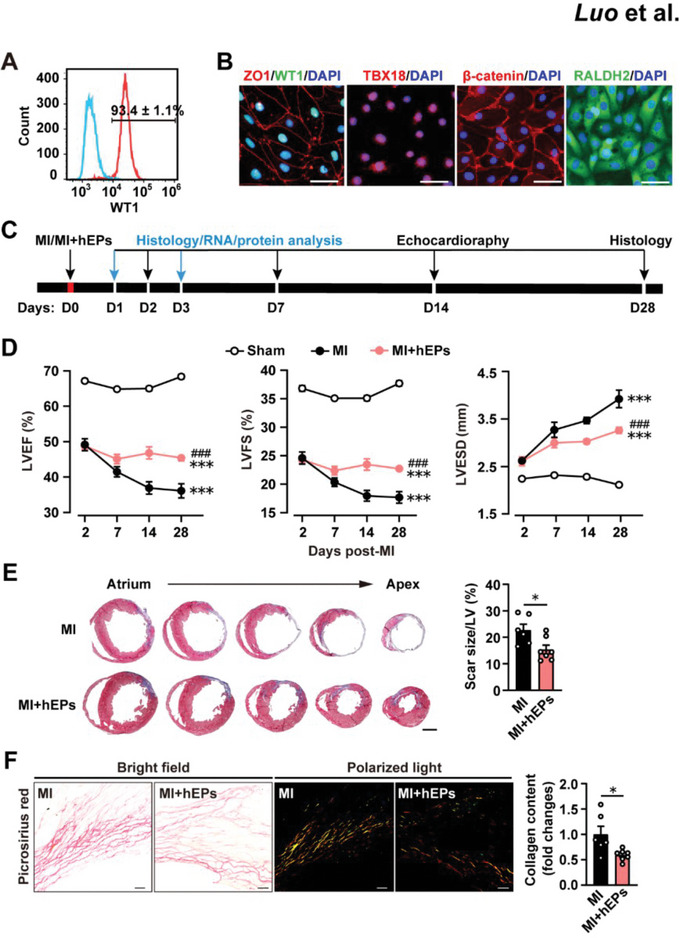
Cardiac reparative effects of human embryonic stem cell‐derived epicardial cells (hEPs) delivered to acutely infarcted mouse hearts. A) Flow cytometry analysis of WT1‐positive hEPs at differentiation day 12. *n* = 4. B) Representative immunocytochemical staining of hEPs at differentiation day 20. *n* = 3. Scale bar: 50 µm. C) Schematic of hEP or vehicle treatment and analysis in the myocardial infarction (MI) hearts. D) Left ventricular ejection fraction (LVEF), LV fractional shortening (LVFS), and LV end‐systolic diameter (LVESD) measured by echocardiography in M‐mode. *n* = 7 each. ^***^ *p* < 0.001 versus the Sham group; ^###^ *p* < 0.001 versus the MI group. E) Representative cross‐sectional images and quantitative analysis of scar size stained with Masson's trichrome at day 28 post‐MI. *n* = 6 to 7. Scale bar, 1 mm. F) Representative images and quantitative analysis of the collagen content in the border zone of infarcted hearts stained with picrosirius red at day 28 post‐MI. *n* = 6 to 7. Scale bar: 50 µm. Data are means ± S.E.M. Two‐way ANOVA followed by Bonferroni's multiple analysis in D. Unpaired student's *t*‐test in E and F. ^*^ *p* < 0.05.

The hESC H7 line (WiCell) was differentiated to hEPs as described previously^[^ ^37^ ^]^ with slight modifications (Figure S1A, Supporting Information). Flow cytometry analysis indicated that ≈93% of the hEPs were positive for Wilm's tumor gene 1 (WT1^+^; **Figure** **1**A), a marker of epicardial cells.^[^ ^38^ ^]^ Consistently, immunocytochemical staining confirmed the expression of WT1, zonula occludens 1 (ZO1), T‐box 18 (TBX18), β‐catenin, and retinaldehyde dehydrogenase 2 (RALDH2) in hEPs (Figure 1B). The EMT property of hEPs was confirmed by the differentiation of hEPs to fibroblasts and smooth muscle cells (SMCs) (Figure S1B, Supporting Information). Immunocytochemical staining of vimentin and discoidin domain receptor tyrosine kinase 2 (DDR2) verified the fibroblast identity of fibroblast growth factor (FGF)‐treated hEPs (Figure S1C, Supporting Information), and the expression of α smooth muscle actin (α‐SMA) and calponin indicated the SMC identity of TGF‐β plus FGF‐treated hEPs (Figure S1D, Supporting Information). These results indicate the EMT property of the hEPs.

### 2.2 Implantation of hEPs Improves Cardiac Function and Reduces Scar Size

To determine whether the implantation of hEPs promotes cardiac repair, we performed MI surgery by permanent ligation of the left anterior descending (LAD) and compared heart function by echocardiography at days 2, 7, 14, and 28 post‐MI among the groups of Sham, MI control with vehicle injection (MI), and MI hearts with the implantation of 5 × 10^5^ hEPs (MI + hEPs) at the acute phase of MI (Figure 1C). The death rates of MI and MI + hEPs mice were comparable during a 28‐day follow‐up period (4 deaths in 22 [18.2%] versus 3 deaths in 18 [16.7%], respectively; *p* > 0.05; Figure S2A, Supporting Information). The body weights of the mice between MI and MI + hEPs groups were also similar (Figure S2B, Supporting Information). The comparable reductions in LV ejection fraction (LVEF) and LV fractional shortening (LVFS), as well as the comparable increase in LV end‐systolic diameter (LVESD) at day 2 post‐MI between the MI and MI + hEPs groups (Figure 1D), indicated effective randomization and relatively comparable infarct sizes among the animals. These parameters worsened in the MI group during 28 days post‐MI, but they were significantly improved in the MI + hEPs group (Figure 1D). Consistently, Masson's trichrome staining analysis showed a decrease of scar size in the MI + hEPs group than that in the MI group at day 28 post‐MI (Figure 1E). The picrosirius red staining with polarized light analysis, a standard method to quantify the collagen content,^[^ ^39^ ^]^ further confirmed the significant decrease of collagen content in the infarcted hearts after hEP implantation (Figure 1F). Taken together, these data indicate the therapeutic benefits of hEPs in the improvement of heart function and prevention of LV remodeling when implanted at the acute phase of MI.

### 2.3 hEPs Reduce Cardiomyocyte Apoptosis and Increase Angiogenesis As Well As Lymphangiogenesis

**Figure 2:**
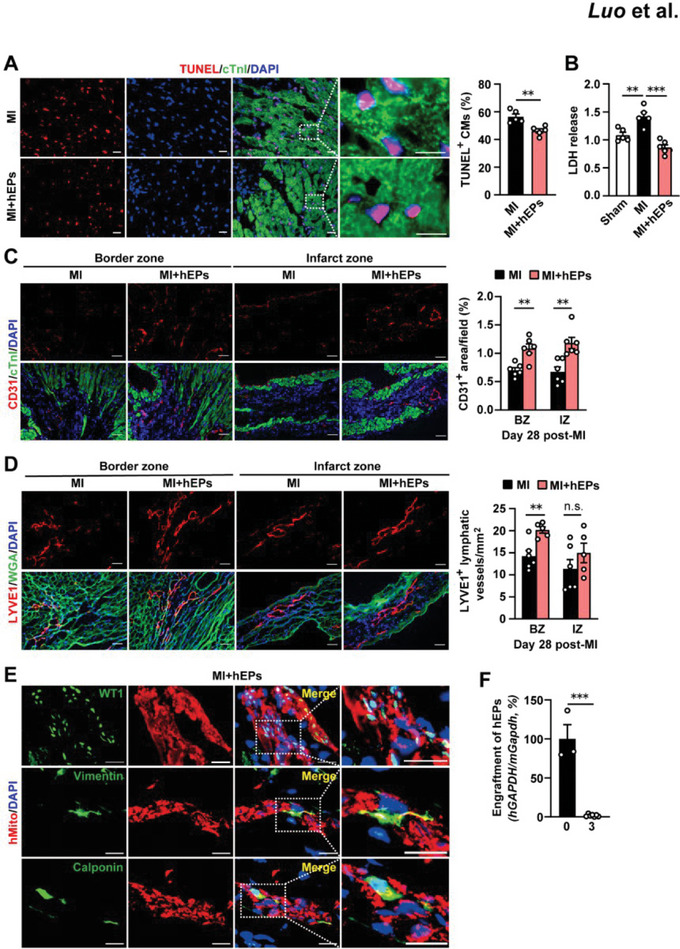
Cardiomyocyte survival, angiogenesis, and lymphangiogenesis regulated by implanted hEPs and the engraftment of hEPs in the infarcted mouse hearts. A) Representative images and quantitative data of immunofluorescent staining for TUNEL^+^ cardiomyocytes (CMs) in the border zone of infarcted hearts at day 3 post‐MI. *n* = 5. Scale bar: 20 µm. B) Lactate dehydrogenase (LDH) activity in mouse serum at day 3 post‐MI. *n* = 5. C,D) Representative images and quantitative data of immunofluorescent staining for CD31^+^ endothelial cells (C) and LYVE1^+^ lymphatic vessels (D) at day 28 post‐MI. *n* = 5 to 6. Scale bar: 50 µm. E) Representative images of co‐immunofluorescent staining for human mitochondria (hMito) with an epicardial marker WT1 or mesenchymal markers (vimentin or calponin) in the infarcted hearts at day 3 post‐MI. Scale bar: 20 µm. 15 sections from 6 hearts were analyzed. F) qRT‐PCR analysis of the transcript level of human GAPDH (hGAPDH) versus mouse Gapdh (mGapdh) in the infarcted LV tissues. The percentage was calculated by normalizing to the average value detected immediately after the hEP injection (day 0). *n* = 3 for day 0, *n* = 7 for day 3. Data are means ± S.E.M. Unpaired student's *t*‐test in (A,C,D,F). One‐way ANOVA followed by Tukey's multiple comparison test in B. ^**^ *p* < 0.01, ^***^ *p* < 0.001. n.s.: no significant difference. BZ, border zone; IZ, infarct zone.

As both increases of cardiomyocyte survival and/or angiogenesis may contribute to the favorable effects of hEPs, we analyzed the number of apoptotic cardiomyocytes, the lactate dehydrogenase (LDH) activity in serum, and the number of platelet‐endothelial cell adhesion molecule 1‐positive (CD31^+^) endothelial cells in the infarcted myocardium. At day 3 post‐MI, hEPs significantly reduced the number of terminal deoxynucleotidyl transferase dUTP nick end labeling positive (TUNEL^+^) cardiomyocytes in the border zones (**Figure** **2**A) and the LDH activities (Figure 2B) compared with the MI group. At day 28 post‐MI, the majority (≈95%) of CD31^+^ cells were negative for the expression of hematopoietic lineage marker CD45 (Figure S3, Supporting Information), enabling the quantitation of vascularization with the number of CD31^+^ cells. An increased number of CD31^+^ endothelial cells in the border and infarct zones of hEP‐treated MI hearts was detected compared with the MI group (Figure 2C). Recent studies indicated that the cardiac lymphatic system is important to the resolution of inflammation and the relief of cardiac edema in the infarcted heart.^[^ ^11^, ^40^ ^]^ We thus examined whether the implantation of hEPs could augment cardiac lymphangiogenesis in the infarcted hearts by analysis of the lymphatic vessels by staining with lymphatic vessel endothelial hyaluronan receptor 1 (LYVE1). While small and sparse lymphatic vessels were detected in Sham hearts (Figure S4, Supporting Information), larger lymphatic vessels with higher density were detected in the border zone of infarcted hearts (Figure 2D). This was further increased in the hEP‐treated MI hearts, though the density of lymphatic vessels in the infarct zones did not show a significant difference between the two groups (Figure 2D). Collectively, the inhibition of cardiomyocyte apoptosis and the promotion of angiogenesis as well as lymphangiogenesis seem to be involved in the reparative effects of hEPs in the infarcted hearts.

To test whether the beneficial effects of hEPs were related to their engraftment and/or EMT property, we performed co‐immunostaining of the human mitochondria protein (hMito), a marker specific to human engrafts, with the epicardial marker WT1 or the mesenchymal markers vimentin and calponin at day 3 post‐MI. Most of the hMito‐positive (hMito^+^) cells detected at day 3 post‐MI were WT1^+^ cells, and a small portion of them expressed vimentin and calponin (Figure 2E). Further, quantitative real‐time polymerase chain reaction (qRT‐PCR) analysis showed a rapid decline of human glyceraldehyde‐3‐phosphate dehydrogenase (hGAPDH) levels in the LV tissues at day 3 post‐MI (Figure 2F). In addition, hMito^+^ cells were hardly detected in the border and infarct zones at day 28 post‐MI (Figure S5, Supporting Information). Thus, EMT only occurs in a small number of implanted hEPs, and paracrine signaling seems to play an important role in the long‐term benefits of hEPs for the infarcted heart.

### 2.4 hEPs Attenuate Inflammatory Responses and Favor the Polarization of Reparative Macrophages

**Figure 3:**
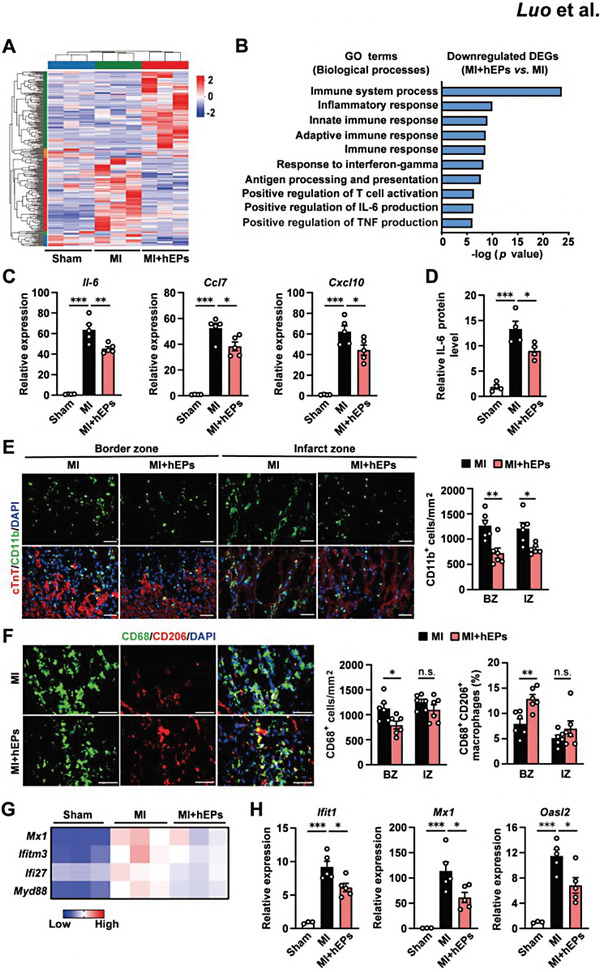
hEPs reduce inflammatory responses and promote reparative macrophage polarization concomitantly with the inhibition of type I interferon (IFN‐I) responses in the mouse LV tissues at day 3 post‐MI. A) RNA sequencing analysis of differentially expressed genes (DEGs, *p* < 0.05 and fold changes ≥ 1.5) in the Sham and infarcted LV tissues with or without hEP treatment. *n* = 3 each. B) Gene oncology (GO) annotation of the DEGs downregulated in the hEP‐treated MI hearts (*p* value < 0.05 and fold change ≥ 1.2). C) qRT‐PCR analysis of the transcript level of cytokine and chemokines. *n* = 4 to 5. D) ELISA analysis of the relative IL‐6 protein levels. *n* = 4. E) Representative immunofluorescent images and quantification of CD11b^+^ cells. *n* = 6. Scale bar, 50 µm. F) Representative images and quantitative data of immunofluorescent staining for CD68^+^ cells and CD68^+^CD206^+^ macrophages in the border zones. *n* = 6. Scale bar: 50 µm. G) The expression of IFN‐stimulated genes (ISGs) in hEP‐downregulated DEGs. H) qRT‐PCR analysis of the transcriptional level of ISGs. *n* = 3 to 5. Data are means ± S.E.M. One‐way ANOVA followed by Tukey's multiple comparison test in (C,D,H). Unpaired student's *t*‐test in (E,F). ^*^ *p* < 0.05, ^**^ *p* < 0.01, and ^***^ *p* < 0.001. n.s.: no significant difference.

To understand the molecular mechanism underlying the reparative effect of hEPs, we analyzed the LV tissues at day 3 post‐MI by RNA sequencing. Hierarchical cluster analysis showed that the hEP implantation‐induced upregulated genes were distinct from those in the Sham and MI groups, and the MI‐induced upregulated transcriptional signatures were reduced by hEPs to a level similar to those in the Sham group (**Figure** **3**A). Gene ontology (GO) analysis of biological processes showed that differentially expressed genes (DEGs) upregulated by hEPs were mainly involved in the regulation of the transcription process, cell differentiation, metabolism, apoptosis, and protein stabilization (Figure S6, Supporting Information). Notably, the top 10 GO terms in hEP‐downregulated DEGs compared to those in the MI group were related to the immune response (Figure 3B; Table S1, Supporting Information). In accordance, qRT‐PCR analysis further confirmed the suppression of MI‐induced increases of pro‐inflammatory factors, including Il‐6, C─C motif chemokine ligand 7 (Ccl7), and C─X─C motif chemokine ligand 10 (C*x*cl10), by hEPs (Figure 3C). Enzyme‐linked immunosorbent assay (ELISA) further confirmed the downregulated protein level of IL‐6 in the infarct and border zones of hEP‐treated hearts at day 3 post‐MI (Figure 3D).

As neutrophils and monocytes/macrophages contribute to cytokine releases and immune responses at the early phase of MI,^[^ ^4^ ^]^ we then analyzed their infiltration in the infarcted heart at day 3 post‐MI. Immunofluorescent staining showed that MI‐induced infiltration of CD11b‐positive (CD11b^+^) neutrophils and monocytes/macrophages was significantly suppressed in the border and infarct zones of hEP‐treated hearts (Figure 3E). Meanwhile, the infiltration of CD68‐positive (CD68^+^) monocytes/macrophages in the border zones but not the infarct zones was significantly reduced by hEPs (Figure 3F). However, the proportion of CD206^+^CD68^+^ reparative macrophages in the border zone of hEP‐treated MI hearts was higher than that in the MI control hearts (Figure 3F), though it was similar in the infarct zones of the two groups (Figure 3F; Figure S7A, Supporting Information). The temporal and regional induction of chemokine (C‐C Motif) receptor 2 (CCR2)^+^ and chemokine (C‐X3‐C) receptor 1 (CX3CR1)^+^ macrophages has been shown to mediate the beneficial effects of adult stem cells in a mouse model of I/R injury.^[^ ^41^ ^]^ We thus examined these subtypes and found that the implantation of hEPs reduced the number of CCR2^+^ macrophages (Figure S7B, Supporting Information) but increased the number of CX3CR1^+^ macrophages (Figure S7C, Supporting Information), compared with those in the MI group at day 3 post‐MI. Taken together, these data indicate the suppression of MI‐induced over‐responsive inflammation and promotion of reparative macrophage polarization by hEPs.

### 2.5 The IFNAR Agonist RO8191 Weakens Anti‐Inflammatory and Myocardial Reparative Effects of hEPs

To identify the molecular mechanisms underlying the regulatory effect of hEPs in MI‐induced inflammatory responses, we further analyzed the downregulated DEGs in the hEP‐treated MI hearts annotated by the GO term innate immune response (Table S1, Supporting Information) and identified a subcluster of ISGs, including Mx1, Ifitm3, Ifi27, and Myd88 (Figure 3G), which function as the downstream executors of IFN‐I signaling for the innate immune response.^[^ ^42^, ^43^ ^]^ Accordingly, qRT‐PCR analysis confirmed the significant downregulation of MI‐increased transcripts of classical ISGs (Ifit1, Mx1, and 2′‐5′ oligoadenylate synthetase like 2 [Oasl2]) in the hEP‐treated LV tissues (Figure 3H).

To test whether the inhibition of IFN‐I responses contributed to the beneficial effects of hEPs, we treated isolated mouse peritoneal macrophages with hEP‐CdM in the presence or absence of IFN‐β stimulation because monocytes/macrophages are primary responders to the IFN‐I signaling and inflammation following MI.^[^ ^36^, ^39^ ^]^ Macrophages can be polarized to a pro‐inflammatory (M1‐like) phenotype, which displays flat and round morphology following lipopolysaccharide (LPS) and IFN‐γ treatments, or to a reparative (M2‐like) phenotype, which displays stretched and elongated morphology following IL‐4 and IL‐13 stimulation.^[^ ^4^, ^44^ ^]^ Morphologically, hEP‐CdM‐treated macrophages were similar to the macrophages polarized by IL‐4/IL‐13 but differed from those polarized by LPS/IFN‐γ or ones treated with the DMEM as a vehicle control, and the hEP‐CdM‐induced alternation of the morphology seemed slightly changed in the IFN‐β‐treated ones (Figure S8A, Supporting Information). qRT‐PCR analysis showed that IFN‐β‐induced increases of the transcript levels of inflammatory *ISGs (Ifit1, Mx1, and Oasl2)* in macrophages were significantly reduced by the hEP‐CdM (Figure S8B, Supporting Information). Concomitantly, the hEP‐CdM markedly suppressed the IFN‐β‐stimulated expression of genes coding proinflammatory factors (Ifn‐β, tumor necrosis factor‐alpha [Tnf‐α], and inducible nitric oxide synthase [(iNos]) but elevated the expression of M2 marker (arginase 1, Arg1) even in the IFN‐β‐treated macrophages (Figure S8B, Supporting Information). These data demonstrate that the hEPs inhibit IFN‐I‐stimulated inflammatory responses and polarize macrophages to the reparative phenotype via paracrine signals.

**Figure 4:**
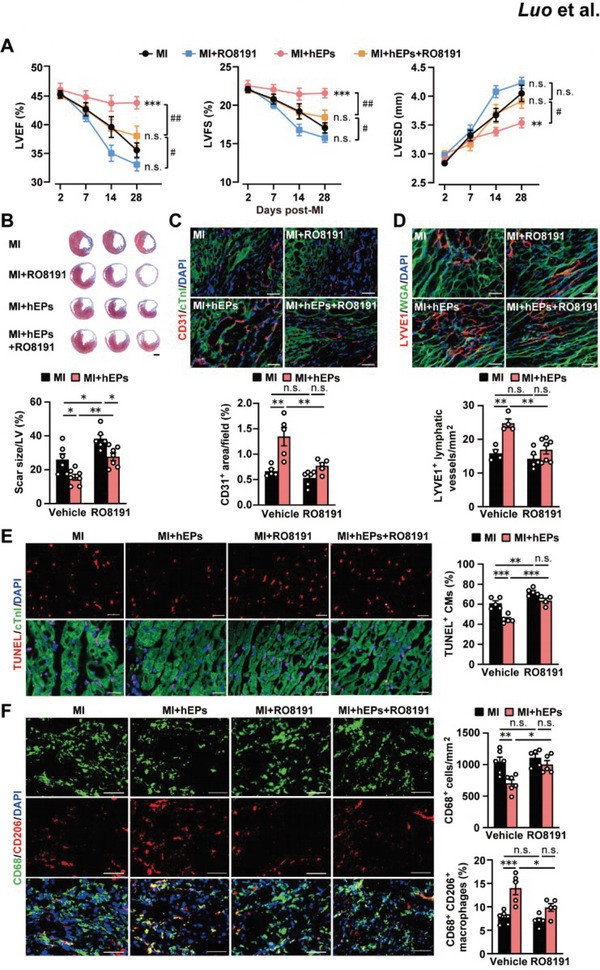
The IFN‐I receptor agonist RO8191 attenuates the anti‐inflammatory and cardiac reparative effects of hEPs. A) Cardiac performance measured by echocardiography. All mice received intragastric administration of 10 mg kg^−1^ RO8191 or vehicle control (corn oil) 30 min before MI surgery. *n* = 6, 8, 6, and 8 in the MI, MI + hEPs, MI + RO8191, and MI + hEPs + RO8191 groups. ^**^ *p* < 0.01, ^***^ *p* < 0.001, and n.s.(no significant difference) versus the MI group. ^#^ *p* < 0.05, ^##^ *p* < 0.01, and n.s. as indicated. B) Representative images and quantitative analysis of scar sizes stained with Masson's trichrome at day 28 post‐MI. *n* = 6 to 7. Scale bar: 1 mm. C,D) Representative images and quantitative data of immunofluorescent staining for CD31^+^ endothelial cells (C, *n* = 5 to 6) and LYVE1^+^ lymphatic vessels (D, *n* = 4 to 7) in the border zones at day 28 post‐MI. Scale bar: 50 µm. E) Representative images and quantitative data of immunofluorescent staining for TUNEL^+^ CMs in the border zones at day 3 post‐MI. *n* = 4 to 5. Scale bar: 20 µm. F) Representative images and quantitative data of immunofluorescent staining for CD68^+^ cells and CD68^+^CD206^+^ macrophages in the border zones at day 3 post‐MI. *n* = 5 to 6. Scale bar: 50 µm. Data are means ± S.E.M. Two‐way ANOVA followed by Bonferroni's multiple analysis in A to F. ^*^ *p* < 0.05, ^**^ *p* < 0.01, and ^***^ *p* < 0.001. n.s.: no significant difference.

Next, we examined whether the IFN‐I signaling is involved in the reparative and inflammatory modulation effects of hEPs by intragastric administration of an IFNAR agonist, RO8191, in mice as reported previously^[^ ^45^, ^46^ ^]^ at 30 min before the implantation of hEPs. RO8191 administration resulted in declining trends in LVEF, LVFS, and LVESD, though the parameters did not show statistical significance compared with the MI group (**Figure** **4**A). Notably, it attenuated the improvement of cardiac function (LVEF and LVFS) and LV dilation (LVESD) by hEPs in the infarcted hearts (Figure 4A). Masson's trichrome staining further confirmed enlarged scar sizes by RO8191 in both MI and hEP‐treated MI hearts (Figure 4B). Concomitantly, the RO8191 treatment inhibited the hEPs‐induced increases in CD31^+^ endothelial cells (Figure 4C) and LYVE1^+^ lymphatic vessels (Figure 4D) in the border zones. Moreover, the reduction of TUNEL^+^ cardiomyocytes by the hEPs was cancelled by the RO8191 treatment at day 3 post‐MI (Figure 4E). Immunofluorescent staining further confirmed that RO8191 inhibited the effects of hEPs on the suppression of monocyte/macrophage infiltration and the promotion of reparative macrophage polarization in the border zones at day 3 post‐MI (Figure 4F). Taken together, these data indicate that hEP‐promoted polarization of reparative macrophages and infarct healing are at least partially associated with the inhibition of IFN‐I signaling.

### 2.6 hEP‐Secreted ITLN1 Interacts With IFN‐β and Inhibits IFN‐β‐Stimulated Responses in Macrophages

**Figure 5:**
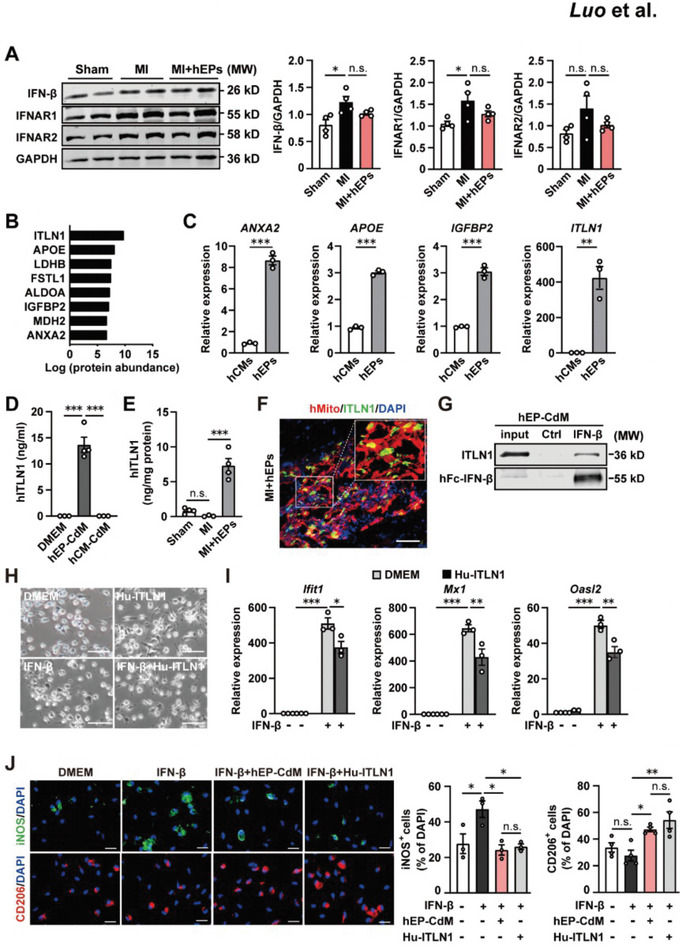
hEPs abundantly secrete ITLN1 and inhibit IFN‐I responses in macrophages. A) Western blotting analysis of IFN‐I signaling‐related proteins in the LV tissues at day 1 post‐MI. *n* = 4. B) Mass spectrometry (MS) analysis revealed top eight enriched proteins in the co‐immunoprecipitated (co‐IP) precipitate of IFN‐β and the proteins in the hEP‐conditioned medium (hEP‐CdM). C) qRT‐PCR analysis of the transcriptional levels of candidates screened by MS in the hESC‐derived cardiomyocytes (hCMs) and hEPs. *n* = 3. D) ELISA analysis of the hITLN1 level in the CdM of hEPs and hCMs. *n* = 3 to 4. E) ELISA analysis of the hITLN1 protein level in the LV tissues at day 1 post‐MI. *n* = 3 to 4. F) Representative image of co‐immunofluorescent staining for hMito and ITLN1 at day 3 post‐MI in the infarcted hearts treated with hEPs. Scale bar: 50 µm. G) Co‐IP analysis showed the interaction between ITLN1 and hFc‐tagged IFNβ (hFc‐IFNβ) in the hEP‐CdM. *n* = 3. H) Representative images showing the morphology of mouse peritoneal macrophages after 24 h with various treatments. *n* = 3. Serum‐free DMEM was used as a vehicle control. The cells were treated with vehicle supplemented with 300 ng mL^−1^ human recombinant (Hu)‐ITLN1 and/or 100 U mL^−1^ IFN‐β. Scale bar: 50 µm. I) qRT‐PCR analysis of ISG mRNAs in mouse peritoneal macrophages after 24 h‐treatment with IFN‐β and/or Hu‐ITLN1. *n* = 3. J) Representative images and quantification of immunofluorescent staining for iNOS^+^ and CD206^+^ macrophages after various treatments. *n* = 3 to 4. Scale bar: 20 µm. Data are means ± S.E.M. One‐way ANOVA followed by Tukey's multiple comparison test in (A,D,E,J). Unpaired student's *t*‐test in (C). Two‐way ANOVA followed by Bonferroni's multiple analysis in (H). ^*^ *p* < 0.05, ^**^ *p* < 0.01, and ^***^ *p* < 0.001. n.s., no significant difference.

Next, we explored how hEPs regulated IFN‐I signaling. As the binding of the IFN‐I ligands to IFNARs initiates the expression of ISGs,^[^ ^33^ ^]^ we examined the protein levels of IFN‐β and its receptors in the infarcted hearts at day 1 post‐MI. While the levels of IFN‐β and IFNAR1 proteins were upregulated following MI, their levels were comparable between the MI and MI + hEP groups (**Figure** **5**A). Meanwhile, the MI‐induced upregulation tendency of IFNAR2 was not significantly altered by the implantation of hEPs (Figure 5A). We then tested whether the paracrine factors secreted from hEPs would interact with IFN‐I ligands and influence the initiation of the IFN‐I signaling. Co‐immunoprecipitation (co‐IP) using hFc‐tagged IFN‐β (hFc‐IFN‐β) with the hEP‐CdM followed by mass spectrometry (MS) analysis identified top eight abundant proteins (Figure 5B), among which the transcript levels of annexin A2 (*ANXA2*), apolipoprotein E (APOE), insulin‐like growth factor binding protein 2 (IGFBP2), and ITLN1 were enriched in the hEPs but not in the hCMs and the abundance of ITLN1 was the highest (Figure 5C). Consistently, the human ITLN1 (hITLN1) protein was easily detected in the hEP‐CdM but not in the hCM‐CdM (Figure 5D). Moreover, the implantation of hEPs markedly increased the protein level of hITLN1 in the LV tissues, whereas a lower level of hITLN1 was detected in the Sham and day 1 post‐MI hearts (Figure 5E). Immunofluorescent staining further confirmed the expression of ITLN1 in the hMito^+^ engrafts at day 3 post‐MI in the infarcted hearts treated with hEPs (Figure 5F). Further, the interaction between ITLN1 and IFN‐β in the hEP‐CdM was confirmed by co‐IP analysis (Figure 5G). We then treated the IFN‐β‐stimulated macrophages with recombinant human ITLN1 protein (Hu‐ITLN1) to determine whether the ITLN1 mimics the effect of hEPs in the suppression of ISGs. The elongated morphology was observed in the Hu‐ITLN1‐treated macrophages compared with that in the DMEM‐treated ones, which was inhibited following the IFN‐β treatment (Figure 5H). In line with the changes in macrophage morphology, IFN‐β‐induced increases in the expression of Ifit1, Mx1, and Oasl2 in macrophages were significantly suppressed by Hu‐ITLN1 (Figure 5I). Supportively, Hu‐ITLN1 and hEP‐CdM treatments decreased the IFN‐β‐upregulated proportion of iNOS^+^ macrophages to a similar level of that in the control group and increased the proportion of CD206^+^ macrophages under IFN‐β stimulation, though the proportion of CD206^+^ cells did not differ between the groups with or without IFN‐β stimulation (Figure 5J). Taken together, the results reveal that hEPs abundantly secrete ITLN1, which interacts with IFN‐β and inhibits IFN‐β‐induced IFN‐I responses as well as M1‐like polarization while promotes reparative polarization in macrophages.

### 2.7 hEPs Promote Infarct Repair Via Secreted ITLN1

**Figure 6:**
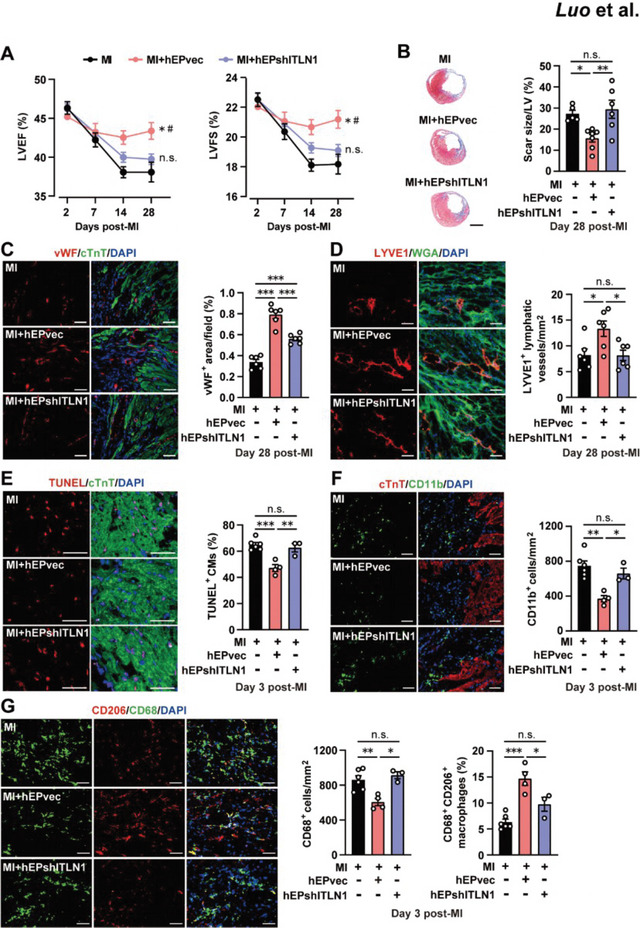
Knockdown of ITLN1 in hEPs weakens their cardioreparative and anti‐inflammatory effects. A) Measurements of cardiac function by echocardiography. *n* = 5, 6, and 7 for the MI, MI + hEPvec, and MI + hEPshITLN1 groups. ^*^ *p* < 0.05, n.s.: no significant difference versus the MI group; ^#^ *p* < 0.05 versus the MI + hEPshITLN1 group. B) Representative images and quantitative analysis of scar sizes stained with Masson's trichrome at day 28 post‐MI. *n* = 5 to 7. Scale bar: 1 mm. C,D) Representative images and quantitative data of immunofluorescent staining for vWF^+^ endothelial cells (C) and LYVE1^+^ lymphatic vessels (D) in the border zones at day 28 post‐MI. *n* = 6. E,F) Representative images and quantitative data of immunofluorescent staining for TUNEL^+^ CMs (E) and CD11b^+^ cells (F) in the border zones at day 3 post‐MI. *n* = 3 to 6. G) Representative images and quantitative data of immunofluorescent staining for CD68^+^ cells and CD68^+^CD206^+^ macrophages in the border zones at day 3 post‐MI. *n* = 3 to 6. Scale bar: 50 µm for all images. Two‐way ANOVA followed by Bonferroni's multiple analysis in (A). One‐way ANOVA followed by Tukey's multiple comparison test in (B–G). ^*^ *p* < 0.05, ^**^ *p* < 0.01, and ^***^ *p* < 0.001; n.s.: no significant difference.

We next sought to address whether hEPs promoted infarct healing via secreted ITLN1 by knockdown of ITLN1 in hEPs using lentivirus‐mediated ITLN1 short hairpin (sh) shRNA. The hEPs transfected with lentivirus packaged with ITLN1 shRNA (hEPshITLN1) showed robust reductions of ITLN1 at both the transcript (Figure S9A, Supporting Information) and protein levels (Figure S9B, Supporting Information). Meanwhile, ITLN1 protein levels in the CdM of hEPshITLN1 were significantly reduced compared with those in the hEPs transfected with lentivirus packaged with vector control (hEPvec) (Figure S9C, Supporting Information). Knockdown of ITLN1 did not affect the proportion of WT1^+^ populations (>95%) in the hEPshITLN1 compared with that in the hEPs and hEPvec (Figure S9D, Supporting Information). In addition, the in vitro EMT ability of hEPshITLN1 characterized by the differentiation of calponin^+^ or α‐SMA^+^ SMCs (Figure S9E, Supporting Information) and DDR2^+^ fibroblasts remained unchanged (Figure S9F, Supporting Information). We then intramyocardially injected hEPvec and hEPshITLN1 at the acute phase of MI. A decreased tendency of hITLN1 protein level at day 1 post‐MI was observed compared with that in the Sham group, whereas the hITLN1 level was significantly elevated in the hEPvec group but it returned to a level similar to the Sham group in the shITLN1‐treated ones (Figure S9G, Supporting Information). Notably, the improvement of LVEF and LVFS by hEPvec in the infarcted hearts disappeared in the hEPshITLN1‐treated group (**Figure** **6**A). Concomitantly, the hEPvec‐reduced scar size was reversed in the hEPshITLN1‐treated group (Figure 6B). Immunohistochemical staining showed that the increased number of von Willebrand factor positive (vWF^+^) endothelial cells (Figure 6C) and the LYVE1^+^ lymphatic vessels (Figure 6D) in the hEPvec‐treated MI hearts were attenuated in the hEPshITLN1‐treated group. Moreover, the decreased number of TUNEL^+^ cardiomyocytes in the hEPvec‐treated MI hearts was reversed in the hEPshITLN1‐treated hearts at day 3 post‐MI (Figure 6E). We further analyzed the impact of ITLN1 knockdown on the hEP‐mediated inflammation modulation. MI‐induced infiltration of CD11b^+^ cells (Figure 6F) and CD68^+^ cells (Figure 6G) was inhibited, whereas the proportion of CD68^+^CD206^+^ reparative macrophages (Figure 6G) was enhanced by hEPvec but not by hEPshITLN1 in the border zone of the infarcted hearts at day 3 post‐MI. Similar effects were observed in the macrophages in vitro, showing that the hEPvec‐CdM decreased the IFN‐β‐upregulated proportion of iNOS^+^ (M1‐like) macrophages and increased the proportion of CD206^+^ (M2‐like) macrophages, whereas these alterations were reversed by the hEPshITLN1‐CdM treatment (Figure S10A, Supporting Information). Further, the MI‐induced elevation of transcript levels of ISGs (Ifit1, Mx1, and Oasl2) at day 3 post‐MI was inhibited by the implantation of hEPvec, whereas the hEPvec‐mediated inhibitory effects were eliminated in the hEPshITLN1 implantation group (Figure S10B, Supporting Information). These results indicate the cardioreparative and anti‐inflammatory effects of hEPs via their secreted paracrine factor ITLN1.

### 2.8 hEPs Preserve Cardiac Function and Limit Adverse Remodeling in a Porcine Model of Reperfused MI

**Figure 7:**
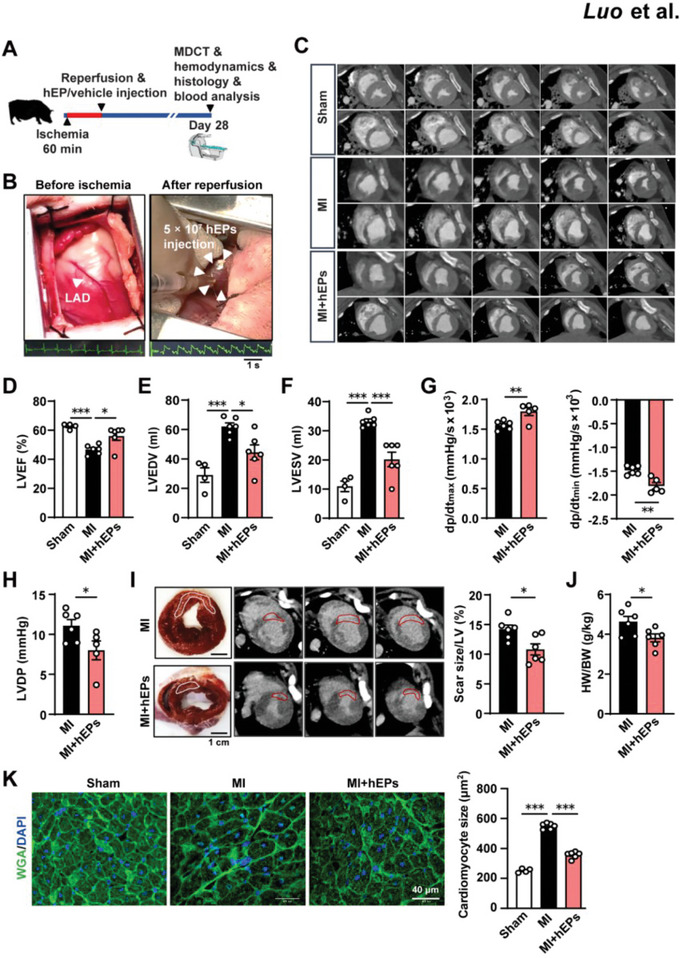
Intramyocardial injection of hEPs improves heart function and reverses cardiac remodeling in the infarcted porcine hearts at day 28 post‐MI. A) Schematic of hEP or vehicle treatment in porcine perfused MI model and analysis. Myocardial injury was induced by LAD occlusion for ischemia (60 min) and reperfusion, followed by intramyocardial injection of 5 × 10^7^ hEPs (MI + hEPs) or with cell suspension vehicle (MI). Heart function was measured by cardiac multi‐detector computed tomography (MDCT). B) Representative photograph of the position for LAD occlusion after thoracotomy (left panel) and for hEP injection into the border zones (white arrowheads) after reperfusion (right panel). Representative normal electrocardiogram (ECG) before the ligation (left bottom) and the ST elevation following reperfusion (right bottom). C) Representative MDCT images of end‐systole (top rows) and end‐diastole (rows below) in each group. D,F) Analyzed data of MDCT for LVEF (D), LV end‐diastolic volume (LVEDV, E), and LV end‐systolic volume (LVESV, F). *n* = 4, 6, and 6 for the Sham, MI, and MI + hEPs groups. G,H) Hemodynamic measurements of maximal LV pressure rising rate (+dp/dt_max_) and minimal LV pressure rising rate (−dp/dt_min_) (G), and LV diastolic pressure (LVDP, H). *n* = 5 to 6. I) Representative heart sections (left) and delayed enhancement (de)‐MDCT images (three in a row, right) of representative porcine hearts. The scar size (within the white line) was calculated as a percentage of the total LV surface area. *n* = 6. J) The heart weight (HW)/body weight (BW) ratio at day 28 post‐MI. *n* = 6. K) Representative images and quantification of immunofluorescent staining for cardiomyocyte cross‐sectional surface area in the border zones. *n* = 4 to 6. 100 cells in two sections from each heart were analyzed. Scale bar: 40 µm. Data are means ± S.E.M. Unpaired student's *t*‐test in (G–J). One‐way ANOVA followed by Tukey's multiple comparison test in (D,E,F,K). ^*^ *p* < 0.05, ^**^ *p* < 0.01, and ^***^ *p* < 0.001.

Next, we evaluated the efficiency of hEPs for infarct repair in a porcine model of reperfused MI induced by LAD occlusion for 60 min followed by reperfusion (**Figure** **7**A) and injection of 5 × 10^7^ hEPs or vehicle into the border zone of the myocardium (Figure 7B). Cardiac function was evaluated at day 28 post‐MI via cardiac multi‐detector computed tomography (MDCT) (Figure 7C) and hemodynamic analyses. hEP implantation prevented MI‐induced decrease of LVEF (Figure 7D) and inhibited MI‐induced upregulation of LV end‐diastolic volume (LVEDV) (Figure 7E) and LV end‐systolic volume (LVESV) (Figure 7F). Meanwhile, hemodynamic measurements indicated that the maximal and minimal rate of pressure change in the ventricle (+dp/dt_max_ and ‐dp/dt_min_) were improved in hEP‐treated MI hearts (Figure 7G). Moreover, the LV diastolic pressure (LVDP) significantly declined in hEP‐treated hearts compared with that in the MI group (Figure 7H). Histological examination and MDCT analysis at day 28 post‐MI showed a significant reduction in the scar size of hEP‐treated hearts (Figure 7I), with concomitant reductions in the heart weight (HW)/body weight (BW) ratio (Figure 7J) and the MI‐induced increase of the cross‐sectional area of cardiomyocytes (Figure 7K). These results suggest the role of hEPs in alleviating the deterioration of systolic function and ventricular remodeling. Meanwhile, heart rates did not alter after hEP implantation at day 28 post‐MI (Figure S11A, Supporting Information). In addition, serum chemistry analysis did not show differences in the indices for renal function (including albumin/globulin [ALB/GLB] ratio, urea, and creatinine), hepatic function (including aspartate aminotransferase [AST], alanine aminotransferase [ALT], γ‐glutamyl transferase [GGT], and alkaline phosphatase [ALP]), or myocardial damage (creatine kinase [(CK]) between the MI and MI + hEP groups at day 28 post‐MI, except for a decrease of alkaline phosphatase (ALP) activity upon the hEP treatment (Figure S11B, Supporting Information). Further examination of the implanted hEPs showed that ≈6 hMito^+^ cells could be detected in each field in the border zones, suggesting low hEP retention at day 28 post‐MI in the infarcted porcine hearts (Figure S11C, Supporting Information).

### 2.9 hEPs Enhance Angiogenesis and Cardiomyocyte Survival As Well As Relieve Inflammatory Responses in Infarcted Porcine Hearts

**Figure 8:**
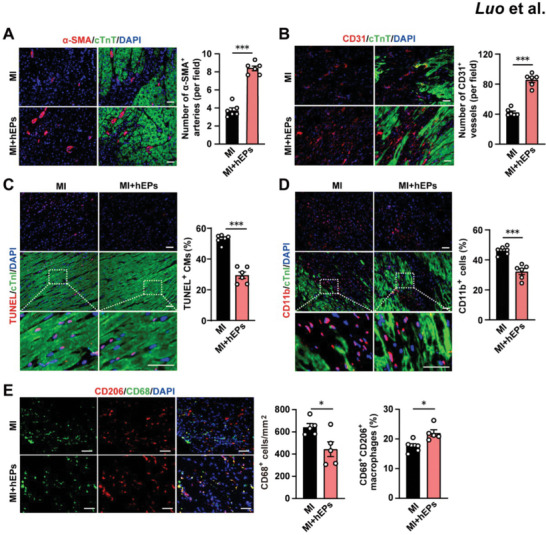
Intramyocardial injection of hEPs promotes angiogenesis, reduces cardiomyocyte apoptosis, and inhibits monocyte/macrophage infiltration in the infarcted swine hearts at day 28 post‐MI. A) Representative images and quantitative data of immunofluorescent staining for α‐SMA^+^ arteries in the border zones. *n* = 6. B) Representative images and quantitative data of immunofluorescent staining for CD31^+^ vessels in the border zones. *n* = 6. C) Representative images and quantitative data of immunofluorescent staining for TUNEL^+^ CMs in the border zones. *n* = 6. D) Representative images and quantitative data of immunofluorescent staining for CD11b^+^ cells in the border zones. *n* = 6. E) Representative images and quantitative data of immunofluorescent staining for CD68^+^ cells and CD206^+^ CD68^+^ macrophages in the border zones. *n* = 5. Scale bar: 50 µm for all images. Unpaired student's *t*‐test in (A–E). ^*^ *p* < 0.05 and ^***^ *p* < 0.001.

Next, we examined whether the implantation of hEPs enhanced endogenous vasculogenic responses in the infarcted porcine hearts at day 28 post‐MI. The number of α‐SMA^+^ arteries (**Figure** **8**A) and CD31^+^ vessels (Figure 8B) in the border zones was increased in hEP‐treated MI hearts compared with that in the MI group. Meanwhile, a fewer number of TUNEL^+^ cardiomyocytes were detected in the border zone of hEP‐treated hearts than in MI hearts at day 28 post‐MI (Figure 8C). Notably, immunofluorescent staining showed significantly decreased infiltration of CD11b^+^ cells in the infarcted myocardium (Figure 8D). Concomitantly, hEPs inhibited the infiltration of CD68^+^ cells and upregulated CD206^+^CD68^+^ reparative macrophages in the border zone of the infarcted hearts at day 28 post‐MI (Figure 8E). Taken together, these data reveal the reparative effects of hEPs in infarcted porcine hearts, which resemble the effects observed in murine hearts, marked by enhanced angiogenesis and cardiomyocyte survival, as well as alleviated inflammatory responses.

## 3 Discussion

This study innovatively explores the function and mechanism of hEPs for infarct healing. We demonstrate that i) hEP implantation at the acute phase of MI improves functional recovery and reduces scar formation in the infarcted mouse hearts. These benefits are accompanied by enhanced cardiomyocyte survival, angiogenesis, and lymphangiogenesis, attenuated monocyte/macrophage infiltration, and increased polarization of reparative macrophages; ii) hEP‐mediated anti‐inflammatory and cardiac reparative effects are counteracted by an IFNAR agonist RO8191; iii) the ITLN1, secreted by hEPs, interacts with IFN‐β and facilitates the hEP‐mediated modulation on IFN‐I responses, inflammation, and infarct healing; and iv) hEPs suppress MI‐induced inflammatory responses and protect infarcted hearts in a clinically relevant porcine model. These findings extend the beneficial effects of hEPs in promoting myocardial infarct repair and reveal previously undiscovered regulatory mechanisms underlying the cardiac reparative effects of hEPs and their paracrine factors.

The first finding in the study is that the implantation of hEPs at the early stage of MI improved cardiac function, prevented LV remodeling, and reduced scar size, accompanied by the promotion of cardiomyocyte survival and angiogenesis in murine and porcine infarcted hearts (Figures 1, 2, 7, and 8). The improvement of cardiac function and pro‐angiogenesis role of hEPs in the mouse infarcted hearts were similar to that mediated by the implantation of EPDCs isolated from human adult atrial tissues.^[^ ^20^, ^21^ ^]^ In addition, we revealed the enhancement of lymphangiogenesis by hEPs. As the lymphatic vasculature is essential for interstitial fluid and immune cell clearance,^[^ ^11^, ^40^, ^47^ ^]^ this effect may be partially involved in the hEP‐mediated benefits for infarct healing. Notably, Bargehr et al.^[^ ^22^ ^]^ did not observe functional improvement and scar limitation in the infarcted rat hearts after intramyocardial injection of hEPs, but they observed larger engraftments and higher proportion of implanted cells expressing mesenchymal marker vimentin (>80%) at day 3 post‐implantation and hEP engraftment at day 28 after implanting at the sub‐acute stage combined with the pro‐survival treatments.^[^ ^22^ ^]^ However, the rapid decline of cell grafts and the small proportion of implanted hEPs expressing mesenchymal markers were detected in ours (Figure 2E,F). The discrepant observations may result from the diverse microenvironmental cues (implantation at the sub‐acute vs acute phase of MI), animals, MI models (I/R model vs permanent ligation of MI model), and cell dosages (5 × 10^6^ vs 5 × 10^5^) between two studies. The reparative effects of hEPs when implanted at the acute phase of MI suggest the early stage of MI as a critical time window for cell therapy. The fast clearance of implanted hEPs in our study may be related to the following factors: i) the acute phase of MI is harsh to the implanted cells. A low cell survival rate (<10%) of the implanted hCMs at day 2.5 post‐MI was observed after implanting the cells to the NOD‐SCID mice at the acute phase.^[^ ^48^ ^]^ At the early stage, the MI induces the recruitment of neutrophils and macrophages.^[^ ^4^, ^23^, ^25^ ^]^ The macrophages were reported to mediate the phagocytosis of the grafted cells in the NOD‐SCID mouse.^[^ ^49^ ^]^ ii) the pretreatment of hEPs with a pro‐survival cocktail in our study seems insufficient to enhance their resistance to stress as long‐term engraftment of hEPs could only be detected in the infarcted NOD‐SCID mouse hearts when cells were pretreated with a pro‐survival cocktail plus heat shock.^[^ ^22^ ^]^ iii) the immune suppression effects are insufficient. The NOD‐SCID mouse is characterized by an absence of functional T cells and B cells, whereas the low activity of natural killer cells can still threaten the survival of the implanted cells.^[^ ^50^ ^]^ iv) The implanted cell dose may affect the retention time. For example, the graft size and retention time are increased along with the increased implantation of hEPs from 2 ×10^6^ to 6 × 10^6^.^[^ ^22^ ^]^ Several strategies for improving the long‐term engraftment have been developed or are under investigation, including enhancement of the stress resistance of hEPs by cell preconditioning or genome editing, and control of immune rejection to the implanted cells, such as using immunosuppressive drugs, biobanking hPSC lines with fine matching human leukocyte antigen (HLA) of the donor and recipient cells, creating immune‐compatible donor hPSC lines, and antibody‐based preconditioning protocol for HLA mismatched cell engraftment.^[^ ^8^, ^50^ ^]^ Notably, both studies provided evidence for the EMT property of implanted hEPs. Whether the improvement of retention and engraftments of hEPs could enhance the EMT of hEPs, and subsequently, contribute to the therapeutic efficacy of infarcted hearts needs to be investigated. Taken together, the short‐time retention and limited EMT of implanted cells suggest the paracrine effects of hEPs as an important mechanism underlying their cardiac reparative effects.

Second, we reveal a favorable modulatory role of hEPs in MI‐induced inflammation and uncover the suppression of IFN‐I signaling underlying the hEP‐mediated immune modulatory and cardiac reparative effects. This is supported by the following evidence: i) hEPs reduced the infiltration of monocytes/macrophages and the level of inflammatory factors, as well as increased polarization of reparative macrophages in the infarcted murine and porcine hearts (Figures 3 and 8; Figure S7, Supporting Information. ii) the hEP‐CdM and the hEP‐secreted ITLN1 inhibited IFN‐β‐induced increases of inflammatory levels, suppressed M1‐like macrophage proportion, and enhanced M2‐like macrophage proportion in isolated macrophages (Figure 5J; Figures S8 and S10A, Supporting Information. iii) Implantation of hEPs inhibited MI‐increased IFN‐I responses, while the hEP‐afforded beneficial effects were dampened by an IFNAR agonist RO8191 (Figure 4). The immune regulatory role of epicardial signals during MI are complex: endogenous C/EBP signals are shown to mediate epicardial activation and promote neutrophil recruitment and inflammatory responses in the infarcted hearts,^[^ ^51^ ^]^ whereas the YAP/TAZ signaling is shown to suppress post‐infarct inflammatory responses through the recruitment of Tregs.^[^ ^52^ ^]^ In the present study, we provide evidence showing that human exogenous epicardial cells suppressed IFN‐I signaling‐mediated inflammation and promoted reparative macrophage shifts in the infarcted hearts. The reparative macrophages mediated the repair of infarcted hearts,^[^ ^53^ ^]^ which were closely associated with their capabilities to reduce apoptotic cardiomyocytes,^[^ ^54^, ^55^ ^]^ promote angiogenesis,^[^ ^29^ ^]^ and lymphangiogenesis.^[^ ^56^, ^57^ ^]^ Based on the different effects of CCR2^+^ and CX3CR1^+^ macrophages on the induction of cardiac fibroblast activity and the reduction of extracellular matrix content in the border zones,^[^ ^41^ ^]^ the hEP‐mediated downregulation of CCR2^+^ subtype versus the upregulation of CX3CR1^+^ subtype in the border zone of the infarcted hearts (Figure S7B,C, Supporting Information) might further explain their enhanced mechanical function against fibrosis and cardiac functional deterioration. It has been shown that the MI‐stimulated IFN‐I signaling induces inflammatory cascades in macrophages through a dsDNA‐sensitive pathway and worsens MI‐induced cardiac injury.^[^ ^35^, ^36^ ^]^ In addition, IFN‐β can block IL‐4‐induced reparative polarization of macrophages by controlling α‐ketoglutarate/succinate metabolism.^[^ ^58^ ^]^ Here, we found that hEPs played an important role in the suppression of IFN‐I signaling‐mediated inflammatory responses and the promotion of reparative macrophage polarization within local niches of the infarcted hearts, associated with the improvement of infarct healing. These findings suggest that the modulation of microenvironment by cell–cell communications critically affects the outcomes of infarcted hearts. However, our data cannot exclude the possibility of inflammatory modulation of hEPs via IFN‐I signaling‐independent pathways in the infarcted hearts, which requires investigation in the future.

Third, we identify ITLN1 as a paracrine factor abundantly secreted from hEPs to interact with IFN‐β and favor cardiac repair. hEP‐secreted hITLN1 interacted with IFN‐β and inhibited IFN‐β‐induced responses in macrophages (Figure 5). hEP implantation significantly upregulated the hITLN1 protein level in the infarcted hearts at day 1 post‐MI, whereas knockdown of hITLN1 in hEPs attenuated their effects on cardiac repair and the inhibition of IFN‐I responses and inflammation (Figure 6). Meanwhile, knockdown of ITLN1 blocked the effects of hEP‐CdM on the inhibition of M1‐like polarization and on the promotion of M2‐like polarization in isolated macrophages (Figure S10, Supporting Information). ITLN1 belongs to adipocytokines.^[^ ^59^ ^]^ Intravenous injection of ITLN1 protein or adenovirus expressing ITLN1 rescues the cardiac functional deterioration by reducing cardiomyocyte apoptosis in a mouse I/R model.^[^ ^60^ ^]^ The latter has been shown to ameliorate unbalanced mitochondrial fusion–fission dynamics and activate mitophagy in mouse MI‐induced heart failure.^[^ ^61^ ^]^ In addition, the suppression of inflammatory responses in macrophages and the promotion of reparative macrophage shifts by hITLN1 have been reported to contribute to the alleviation of atherosclerosis and metabolic syndrome.^[^ ^62^, ^63^ ^]^ Our observations here uncover the novel role of hEP‐secreted ITLN1 in the modulation of angiogenesis, lymphangiogenesis, IFN‐β‐mediated inflammatory responses in macrophages, cardiomyocyte survival, and cardiac repair. Previous studies have shown that hEP‐secreted fibronectin can promote hCM maturation and proliferation when co‐cultured or co‐implanted with hCMs.^[^ ^22^ ^]^ The CdM^[^ ^18^ ^]^ and extracellular vesicles (EVs)^[^ ^19^ ^]^ from hEPDCs were reported to promote angiogenesis and enhance cardiomyocyte proliferation in injured neonatal hearts and hCM‐engineered heart tissue, respectively. These findings, together with our observations, suggest that the paracrine factors secreted from hEPs are important sources for the modulation of the local inflammatory niche, extracellular matrix, and functions of neighboring cells, leading to the repair and regeneration of infarcted hearts. Moreover, a cell interaction mechanism has been suggested as hEPs can promote cardiomyocyte differentiation of hPSC‐derived cardiac progenitor cells when in direct contact with each other.^[^ ^64^ ^]^ Considering the ability of implanted hEPs to continuously produce paracrine factors and exert effects via EMT/differentiation or direct interaction with other cell types, it is valuable to determine whether the efficacy of hEPs and their CdM/EVs in cardiac repair is comparable upon the improvement of cell retention in future. Further, the interaction between ITLN1 and IFN‐β identified in this study reveals a novel modulatory pattern for IFN‐β‐mediated signaling, which provides new insights into the regulation of IFN‐β‐induced signaling besides the transcriptional regulation of IFN‐I genes by transcription factors (i.e., IRF3, IRF7, NF‐κB, and activator protein 1) and epigenetic modifications.^[^ ^65^, ^66^ ^]^

There are limitations in this study. The effects of hITLN1 on cardiac repair and inflammatory regulation in the infarcted hearts were only examined by the treatment of macrophages with Hu‐ITLN1 in vitro and the implantation of hEPshITLN1 in vivo. Whether and how the direct injection of Hu‐ITLN1 affect inflammation and infarct healing need to be investigated in future. Moreover, immunosuppressants (cyclosporine and methylprednisolone) were applied according to previous reports.^[^ ^7^, ^67^, ^68^ ^]^ However, we did not measure the real‐time plasma concentration of immunosuppressants in the present study. This needs to be performed in future studies to identify an optimal dosage of cyclosporine or the addition of other immunosuppression drugs to minimize the immune rejection and accomplish a higher cell engraftment rate in swine.

## 4 Conclusion

In summary, we report the reparative effects of intramyocardial implantation of hEPs at the acute phase of MI in murine and porcine infarcted hearts. The beneficial effects of hEPs for recovery of cardiac function and reduction of fibrosis are supported by enhanced reparative macrophage polarization, decreased inflammatory responses in monocytes/macrophages, and improved cardiomyocyte survival, angiogenesis, as well as lymphangiogenesis. Further, we reveal a novel mechanism underlying the reparative effects of hEPs via the secreted paracrine factor, which is associated with the interaction between ITLN1 and IFN‐β, as well as the suppression of IFN‐I signaling. These findings uncover new functions and mechanisms involving paracrine factor‐mediated cell–cell communications during infarct healing and provide a therapeutic option for ischemic myocardial repair.

## 5 Experimental Section

### hESC Culture and Differentiation

The hESCs (H7, WiCell) were cultured on Matrigel (Corning)‐coated surfaces in mTeSR1 medium (StemCell Technologies) as previously described.^[^ ^69^, ^70^ ^]^ The hESCs were differentiated into hEPs as described previously^[^ ^37^, ^71^ ^]^ with slight modifications (Figure S1A, Supporting Information). Briefly, hESCs were plated onto Matrigel‐coated plates at a density of 25 000 cells cm^−2^ in mTesR with 10 µm Y‐27632 (a ROCK inhibitor, StemCell Technologies). When the hESCs reached 85% confluence, 6 µm CHIR99021 (StemCell Technologies) was added to the chemically defined medium (CDM), which contained RPMI‐1640 (Gibco), 213 µg mL^−1^ L‐ascorbic acid 2‐phosphate (Sigma–Aldrich), and 2 mg mL^−1^ bovine serum albumin (BSA, Sigma–Aldrich) to initiate the differentiation. At day 2, the medium was changed to CDM supplement with 5 µm IWR‐1 (Merck). At day 4, the differentiating cells were digested with Accutase (Stem Cell Technologies) for 5 min and then plated at the density of 1:24 onto 0.1% gelatin‐coated surfaces in the LasR medium (advanced DMEM/F12 medium (Gibco) supplement with 1×GlutaMax (Gibco) and 100 µg mL^−1^ ascorbic acid (Sigma–Aldrich) with 10 µm Y‐27632. 24h later, the cells were incubated with 3 µm CHIR99021 in the LasR medium for 2 days; and then, the LasR medium was changed every 2 days until passaging on day 12. The hEPs were then dissociated with 0.05% Trypsin‐EDTA (Thermo Fisher) for passaging, cryopreservation, and analysis.

To initiate the EMT of hEPs, cells at differentiation day 15 were plated onto the Matrigel‐coated plates at the density of 6 × 10^5^ cells cm^−2^ in LasR medium with 10 µm Y‐27632 (Figure S1B, Supporting Information). After 24 h cultivation, the medium was changed to the LasR medium supplement with 10 ng mL^−1^ FGF or 5 ng mL^−1^ TGF‐β for the differentiation of fibroblasts and smooth muscle cells, respectively. After a 3‐day incubation, the medium was changed to LasR medium supplement with 10 ng mL^−1^ FGF for another 3‐day cultivation. The differentiated cells were replated onto Matrigel‐coated coverslips for further analysis.

### Flow Cytometry Analysis

Cells were analyzed by flow cytometry as described previously.^[^ ^69^, ^70^ ^]^ Briefly, the differentiated hEPs were collected after dissociation with 0.05% Trypsin‐EDTA for 5 min. The cells were fixed and permeabilized with the Foxp3 Staining Buffer kit (Invitrogen, 00‐5523‐00) for 30 min, followed by incubation with the primary antibody (anti‐WT1, Abcam, ab89901) for 1 h at room temperature (RT) and the PE‐conjugated secondary antibody (Biolegend, 1:400) for 1 h at 4 °C. Cells were then analyzed with a flow cytometer (Beckman CytoFlex) and quantified with FlowJo software.

### MI Models and Study Designs

Adult male NOD‐SCID mice were purchased from the Shanghai SLAC in Laboratory Animal Co. Ltd., Shanghai, China. Bama swine (female, 20 kg, 16–20 weeks old) were purchased from Shanghai Jiagan Laboratory Animal Co., Ltd. The experimental procedures involving mice were approved by the Institutional Animal Care and Use Committee (IACUC) of the Shanghai Institute of Nutrition and Health, CAS (Approval Number: SIBS‐2019‐YHT‐1), in accordance with the Guidelines for the Care and Use of Laboratory Animals published by the U.S. NIH; and the procedures and protocols for pigs were approved by the IACUC of Shanghai Pulmonary Hospital, School of Medicine, Tongji University (Approval Number: K19‐147). To induce mouse MI model, mice around 12‐weeks old were subjected to LAD coronary artery ligation, while mice in the Sham group had a loose suture as described previously.^[^ ^4^, ^72^ ^]^ Briefly, the mice were anesthetized via intraperitoneal injection of 50 mg kg^−1^ sodium pentobarbital and ventilated using a rodent ventilator (DW‐3000B) at a frequency of 90 bpm and a stroke volume of 100 mL. The hearts were exposed, and the LAD was ligated at the ≈2–3 mm on the lower edge of the left auricle; then, the mice were randomly divided into the following groups: i) for functional evaluation of hEPs in MI hearts (Sham, MI, and MI + hEPs); ii) for the examination of the IFN‐I signaling involvement in MI hearts treated with hEPs (MI, MI + RO8191, MI + hEPs, and MI + RO8191 + hEPs); and iii) for the contribution of ITLN1 to the benefits of hEPs in MI hearts (MI, MI + hEPvec, and MI + hEPshITLN1). Before implantation, the hEPs at differentiation day 16 were collected and washed twice with DMEM (high glucose, Thermo Fisher). Cell suspensions at the concentration of 2.5 × 10^7^ cells mL^−1^ were prepared with high‐glucose DMEM supplement with pro‐survival cocktails (100 ng mL^−1^ insulin‐like growth factor 1, 200 nm cyclosporine A, 100 µm ZVAD‐FMK, 50 nm Bcl‐XL, and 50 µm pinacidil). Cells (5 × 10^5^ cells in 20 µL) were intramyocardially injected into two sites in the border zone of the MI hearts (10 µL per site) just after the ligation of LAD. Similarly, the vehicle control at the same volume was intramyocardially injected in the MI group. RO8191 (APExBIO) was diluted with corn oil and intragastrically administrated at 10 mg kg^−1^ within 30 min before MI injury. The control group was delivered with corn oil with the same protocol. To induce the reperfused porcine MI model, the myocardial infarction was induced by LAD occlusion for 60 min as described previously.^[^ ^7^, ^73^ ^]^ Briefly, animals were anesthetized with 2% isoflurane and maintained with a respirator. During the operation, vital signs, including electrocardiogram (ECG), blood pressure, arterial oxygen saturation, and body temperature, were monitored. Then, the heart was exposed, and the root of the first and second diagonal coronary arteries from the LAD coronary artery was occluded for 60 min. After reperfusion, the animals in the MI + hEPs group received 5 × 10^7^ hEPs (in 1 mL) by intramyocardial injection in five sites around the border zone, while the MI control group received cell suspension vehicle (high‐glucose DMEM supplement with pro‐survival cocktails). Animals in the Sham group underwent all surgical procedures for MI induction except for LAD occlusion. Animals in all treatments received cyclosporine (15 mg kg^−1^ per day with food) and methylprednisolone (1.5 mg kg^−1^ per day with food) for immunosuppression.

### Echocardiography

The cardiac contractile function and structure were measured as reported previously.^[^ ^4^, ^13^, ^72^ ^]^ Mice were anesthetized with 1.5% isoflurane and fixed after removing the chest hair. The M‐mode echocardiography was performed using the Vevo 2100 imaging system with a 400‐MHz transducer (Visual Sonics Inc., Canada). The LVEF, LVFS, and LVESD were calculated.

### MDCT

Cardiac contractile function and infarct size were measured by analyzing the MDCT images at day 28 post‐MI as previously described.^[^ ^74^ ^]^ Briefly, animals were anesthetized with 2% isoflurane and scanned via an Aquilion ONE TSX‐301C MDCT scanner (Canon Medical). Iopamidol (Bracco Sine, Iopamiro 370) was injected through the auricular vein for MDCT image acquisition. Dual phase ECG gated scan was performed after injection. In the first phase, the myocardial perfusion model and auto trigger scan were used. At last, the de‐MDCT images were acquired with a myocardial delay model 7 min after contrast delivery based on various parameters (gantry rotation time = 275 ms, detector collimation = 0.5 mm × 320, isotropic voxels = 0.5 × 0.5 × 0.5 mm^3^, tube voltage = 100 kV, and tube current = 400 mA). After reconstruction in the optimal phase by dual scanning, images were reformatted to a suitable position for myocardial perfusion and scar assessment (de‐MDCT images). Then, the LVEF, LVESV, and LVEDV were calculated via Vitrea 2 computer workstation (Vital Images). The scar size was calculated as scar area/total LV area × 100%. For each heart, the average value from six cross‐sections of the MDCT images was used for statistical analysis.

### Hemodynamic Assessments

After anesthetization, a polyvinyl chloride catheter was inserted into the LV lumen via the ascending aorta as previously described.^[^ ^7^, ^73^ ^]^ The pressures of the aortic and LV were monitored based on a PowerLab system (AD Instrument), and the LVDP, dP/dt_max_, and dP/dt_min_ were measured at day 28 post‐MI. Then, the blood samples were drawn, and blood chemistry analysis was performed by the Hangzhou Adicom Medical Laboratory Center Co., LTD.

### Analysis of Serum LDH Activity

The sample preparation and LDH activity measurements were performed as previously reported.^[^ ^13^, ^72^ ^]^ Briefly, the blood in the LV was collected from anesthetized mice at day 3 post‐MI. Serum was extracted from the blood supernatant after centrifuging at 3000 rpm for 10 min. The LDH level was measured according to the supplier's instructions (Beyotime). The serum sample (5 µL, diluted into 50 µL with ddH_2_O) and reaction solution (60 µL) were added sequentially into the 96‐well plate. After 30 min incubation at RT, the absorbance was recorded at 490 nm by the Synergy HT microplate reader (BioTek).

### Sample Preparations for Sections

Hearts were quickly removed from anesthetized mice and then washed with cold PBS. For mouse hearts, LV tissues from the point of ligation to the apex were embedded in optimal cutting temperature compound (OCT, SAKURA) for frozen sections. Transversal sections (6 µm) were prepared at 400 µm intervals. For porcine hearts, the apex to the base of the LV wall was uniformly cut into five rings (R1 to R5) after being cleaned in a cryogenic saline solution. Each ring was cut into samples representing infarct, border, and remote zones before being embedded into OCT and sectioned.

### Masson's Trichrome Staining

The scar size was analyzed by Masson's trichrome staining according to the manufacturer's instructions (Solarbio). The scar size was calculated as the percentage of the total scar area versus the LV area as reported previously.^[^ ^4^, ^72^ ^]^ For each heart, five sections (from the point of ligation to the apex of the heart) were quantified with ImageJ, and the mean value was taken as the scar area.

### Picrosirius Red Staining

Following the manufacturers’ instructions (SenBeiJia Biological Technology), the frozen sections were incubated in 4% PFA for 15 min, next in picrosirius red staining solution for 1 h, and then in 0.5% of acetic acid solution for 3 min. After hematoxylin staining for 10 min, the sections were dehydrated and mounted using the resinene. Collagen content was assessed using polarized light microscopy (Carl Zeiss) equipped with the Analyzer module DIC ACR P&C as described.^[^ ^39^ ^]^ The collagen fraction in two sections for each heart was quantitated by ImageJ.

### Immunocytochemical/Immunofluorescent Staining

For immunocytochemical staining, cells were fixed with 4% PFA (Sigma), permeabilized with 0.4% Triton X‐100 (Sigma), and then incubated with the primary antibodies (WT1, Abcam, ab89901; ZO1, Millipore, 339100; TBX18, Santa Cruz, sc‐514486; β‐Catenin, BD, 610154; RALDH2, Sigma, HPA010022; vimentin, CST, 5741S; DDR2, Abcam, ab63337; α‐SMA, Abcam, ab5694; calponin, Abcam, ab46794; iNOS, Abcam, ab129372) at 4 °C overnight. For immunofluorescent staining, the frozen sections were brought to RT for 15 min and washed with PBS twice. The sections were fixed with 4% PFA, permeabilized with 0.4% Triton X‐100, and stained with anti‐CD31 (Abcam, ab28364), vWF (Dako, A0082), LYVE1 (R&D systems, AF2125), CD11b (Abcam, ab8878), CD68 (Abcam, ab53444; Immunoway, YM3050), CD206 (Abcam, ab64693), CX3CR1(ProteinTech, 13885‐1‐AP), CCR2 (Abcam, ab273050), CD45 (ProteinTech, 20103‐1‐AP), WT1 (Abcam, ab89901), cardiac troponin I (cTnI) (Abcam, ab47003), cardiac troponin T (cTnT) (Abcam, ab8295), human mitochondria (hMito) (Millipore, MAB1273), and FITC‐conjugated WGA (Sigma, L4895) antibody. The Alexa 488‐ or 555‐conjugated secondary antibodies were incubated for 1 h in the dark. DAPI was used for nuclei staining. To evaluate the apoptosis at day 3 post‐MI, the in situ Cell Death Detection Kit (Roche, 11684817910) based on the TUNEL staining, was used. The index of apoptosis was quantified with ImageJ and calculated as the number of TUNEL^+^ cells /total number of DAPI^+^ nuclei × 100%. All samples were imaged with a Carl Zeiss microscope and processed with ZEN software. Images from two to three sections were analyzed by ImageJ for each heart.

### Western Blot Analysis

To analyze the protein levels, the border and infarct zones of the infarcted hearts or the cells were collected and quickly frozen in liquid nitrogen as reported previously.^[^ ^4^, ^13^, ^72^ ^]^ To extract the proteins, samples were homogenized with a homogenizer in RIPA lysis buffer containing 20 mm Tris‐HCl (pH 7.4), 1% Triton X‐100, 1% deoxycholate, 10% glycerinum, 150 mm NaCl, 2.5 mm EDTA, and 1 mg mL^−1^ protease inhibitor cocktail on ice. The samples were placed onto the ice for 30 min and then centrifuged at 12 000 × *g* for 20 min. The supernatant was collected, and the concentration of proteins was analyzed with a protein quantitation kit (Thermo Fisher, 23223). The loading buffer was added to the proteins, and the samples were boiled at 96 °C for 10 min. For immunoblotting analysis, the proteins (≈30–50 µg) were loaded into the PAGE‐SDS gels. The proteins were separated and transferred to the nitrocellulose membrane and incubated with specific antibodies against IFN‐β (Abclonal, A1575), IFNAR1 (Sino biotechnology, 50469‐RP01), IFNAR2 (Abclonal, A1769), and GAPDH (Proteintech, HRP‐60004) overnight at 4 °C. IRDye 680LT or IRDye 800LT fluorescent secondary antibodies (Li‐COR Bioscience, 926–32212) were used for detection. The Odyssey Infrared Imager (Li‐COR Biosciences) was used to visualize the bands, and the integrated density of the gray value was measured by ImageJ.

### qRT‐PCR

Total RNAs from the LV tissues (including the infarct and border zones) and the cells were extracted with Trizol (Invitrogen) and RNAprep Pure Micro Kit (TIANGEN BIOTECH, DP420), respectively. The cDNA was synthesized with the ReverTra Ace qPCR RT Kit (TOYOBO, KR116), as previously described.^[^ ^4^, ^13^ ^]^ qRT‐PCR was performed with the SYBR Green qPCR Master Mix (Roche, 4913914001) and detected with the VIIA 7 Real‐Time PCR System (ABI). The transcript level of genes was calculated as 2^(−△△Ct)^, in which △Ct refers to the amplification cycles versus GAPDH, and the △△Ct refers to the △Ct versus the control group. The primers are listed in Table S2, Supporting Information.

### RNA Sequencing Assay

Three replicates from the Sham, MI, and MI + hEPs groups at day 3 post‐MI were used for RNA extraction. The RNA sequencing was carried out with the Illumina Hiseq sequencing platform by Majorbio Company. RNASeq data sets were mapped using the *Mus musculus* genome (http://www.ensembl.org/Mus_musculus/Info/Index). The data were analyzed through the free online platform of Majorbio Cloud Platform (www.majorbio.com). GO analysis was performed with DAVID Bioinformatics Resources (https://david.ncifcrf.gov/).

### Preparation of the hEP‐CdM

The hEPs were seeded onto the 10 cm dishes at a density of 3.5 × 10^4^ cells cm^−2^. After culturing for 24 h, cells were washed with PBS twice, and the medium was changed to high‐glucose DMEM. The hEP‐CdM was collected 48 h later and the cell debris was removed by centrifugation at 2000 g for 5 min.

### Co‐IP Assay

The hEP‐CdM was concentrated 30‐fold with the ultrafiltration centrifuge tube (3 kDa, Millipore) by centrifugation at 4000 g for 1 h. The concentration of proteins was detected with the bicinchoninic acid method. The proteins (≈500 µg) were incubated with or without mouse hFc‐tagged IFN‐β (Sino biotechnology, 1:1000) with end‐over‐end mixing at 4 °C overnight. As previously described,^[^ ^75^ ^]^ the co‐IP was performed using protein G Magnetic Beads (GE health), and the reaction mixtures were mixed with beads (20 µL) at 4 °C for 4 h. The immune‐precipitates were separated from the supernatant using a magnetic frame for magnetic beads and washed five times in IP buffer. The beads were boiled in 1.5× loading buffer at 96 °C for 10 min, and the eluted proteins were collected.

### Silver Staining and MS Analysis

The proteins were loaded into 12% SDS‐PAGE gel. After finishing the protein separation, the gel was fixed overnight and silver staining was performed according to the manufacturer's instructions (Beyotime). The MS was performed by Applied Protein Technology (Shanghai) based on the Q Exactive (Thermo Fisher) and Easy‐nLC 1000 (Thermo Fisher) platforms. The alignment of peptides and proteins was analyzed by the MASCOT software. Candidate proteins were selected based on molecular weight and abundance.

### ELISA

The concentration of hITLN1 was measured using a hITLN1 ELISA kit (Elabscience, E‐EL‐H2028c). For the detection of hITLN1 in LV tissues, proteins were extracted as described above, and the concentration was normalized to 1800 µg mL^−1^ with RIPA lysis buffer. For analysis, samples (100 µL) were added into the coated 96‐well plates, followed by incubation with the primary and HRP‐labeled secondary antibodies. Recombinant hITLN1 was reconstituted and serially diluted to make a standard curve. The plate was analyzed at 450 and 600 nm with the Synergy HT microplate reader (BioTek).

### Isolation and Culture of Peritoneal Macrophages

Peritoneal macrophages from C57BL/6J mice were harvested under aseptic conditions after intraperitoneal injection with 3% thioglycollate (Sigma–Aldrich) for 3 days as previously described.^[^ ^4^ ^]^ Red blood cells were removed with erythrocyte lysis buffer (150 mm NH_4_Cl in 10 mm Tris‐HCl, pH 7.2). The cells were resuspended with RPMI‐1640 supplement with 10% FBS and 1% P/S solution and seeded onto 24‐well plates at the density of 1 × 10^6^ cells mL^−1^. The medium was changed after 4 h of cultivation, and the cells were cultured for 24 h before being treated with hEP‐CdM, Hu‐ITLN1 (Novoprotein or R&D systems, 300 µg mL^−1^), IFN‐β (PBL Assay Science, 100 U mL^−1^), IFN‐β + hEP‐CdM, or IFN‐β + Hu‐ITLN1 in serum‐free DMEM. Cells were collected and analyzed after 24 h treatment.

### Plasmid Construction and Lentivirus Packaging

The shRNA targeting the human ITLN1 genome was designed using the TRC shRNA Design Process website.^[^ ^76^ ^]^ Briefly, the cDNA of shRNA targeting human ITLN1 was synthesized through the annealing process (Beyotime) using the forward and reverse primer sequences of the shRNA (Table S2, Supporting Information). Then, the synthesized cDNA was subcloned into a PLKO.1‐Empty‐Puro SHC001 plasmid (Sigma) between the EcoRI and BshTI restriction endonuclease sites. The plasmid was then verified by Sanger sequencing. The PLKO.1‐Empty‐Puro SHC001 plasmid was used as vector control. For lentivirus packaging, the plasmids were transfected into 293FT cells (Invitrogen) combined with the psPAX2 and pMD2.G plasmids using the lipofectamine 3000 reagents (Invitrogen). After cultivation for 24 h, the medium was changed to DMEM supplement with 10% FBS. The supernatant was collected at 48 and 72 h post‐transfection, and the lentivirus particles were concentrated via ultracentrifugation at 130 000 × g for 2 h. The vector control and *ITLN1* shRNA lentivirus particles were transfected into the hEPs at differentiation day 11 and removed 24 h later. After another 4 days of cultivation, cells were collected for analysis or implantation. For the collection of CdM from hEPvec and hEPshITLN1, cells were washed with PBS twice at day 3 post‐lentivirus transfection, and the medium was changed to high‐glucose DMEM for 2‐day cultivation. The CdM was collected and the cell debris was removed by centrifugation.

### Statistical Analysis

The data was presented as mean ± S.E.M. Unpaired student's *t*‐test was used for two‐group comparison. One‐way analysis of variance (ANOVA) followed by Tukey's multiple comparison test was used for the comparison among groups of more than two as well as the analysis of hITLN1 protein in hEPshITLN1 and the RO8191 treatment groups. Two‐way ANOVA followed by Bonferroni's multiple analysis was used for the analysis of heart function, angiogenesis, immune cell infiltration, and primary mouse macrophages. The statistical analysis was conducted using GraphPad Prism 8.0. *p* < 0.05 was considered to be statistically significant.

## Conflict of Interest

The authors declare no conflict of interest.

## Author Contributions

X.‐L.L. and Y.J. contributed equally to this work. X.‐L.L., Y.J., and H.‐T.Y. designed the research. X.‐L.L. and Y.J. performed most experiments, analyzed data, and wrote the manuscript. Q.L., and X.‐J.Y. contributed to mouse experiments. X.‐L.L., Y.J., T.M., and H.C. contributed to large animal experiments. M.‐X.K. participated in Masson's trichrome staining and statistical analysis. P.Z. contributed to cell culture and experimental design. J.‐L.T. contributed to technical support and manuscript revision. Y.‐S.G. analyzed part of the porcine heart function. L.W. provided some suggestions during this revision and provided financial support for part of the added new experiments. L.G. designed and supervised the large animal experiments, revised the manuscript, and provided financial support. H.‐T.Y. supervised the study, analyzed data, wrote and finally approved the manuscript, and provided financial support.

## Supporting information

Supporting Information

## Data Availability

The data that support the findings of this study are available from the corresponding author upon reasonable request.
