# Supplementary material for: hESC‐Derived Epicardial Cells Promote Repair of Infarcted Hearts in Mouse and Swine
Source: Adv Sci (Weinh). 2023 Jul 28;10(27):2300470. doi: 10.1002/advs.202300470 (PMC10520683; doi:10.1002/advs.202300470)
Supplement: Supplementary file 1 — Supporting Information [file ADVS-10-2300470-s001.pdf]

ADVANCED SCIENCE
Open Access

Supporting Information

for *Adv. Sci.*, DOI 10.1002/advs.202300470

hESC-Derived Epicardial Cells Promote Repair of Infarcted Hearts in Mouse and Swine

Xiao-Ling Luo, Yun Jiang, Qiang Li, Xiu-Jian Yu, Teng Ma, Hao Cao, Min-Xia Ke, Peng Zhang, Ji-Liang Tan, Yan-Shan Gong, Li Wang, Ling Gao and Huang-Tian Yang**

Supporting Information

hESC-Derived Epicardial Cells Promote Repair of Infarcted Hearts in Mouse and Swine

Xiao-Ling Luo[†], Yun Jiang[†], Qiang Li, Xiu-Jian Yu, Teng Ma, Hao Cao, Min-Xia Ke, Peng Zhang, Ji-Liang Tan, Yan-Shan Gong, Li Wang, Ling Gao[*], Huang-Tian Yang[*]

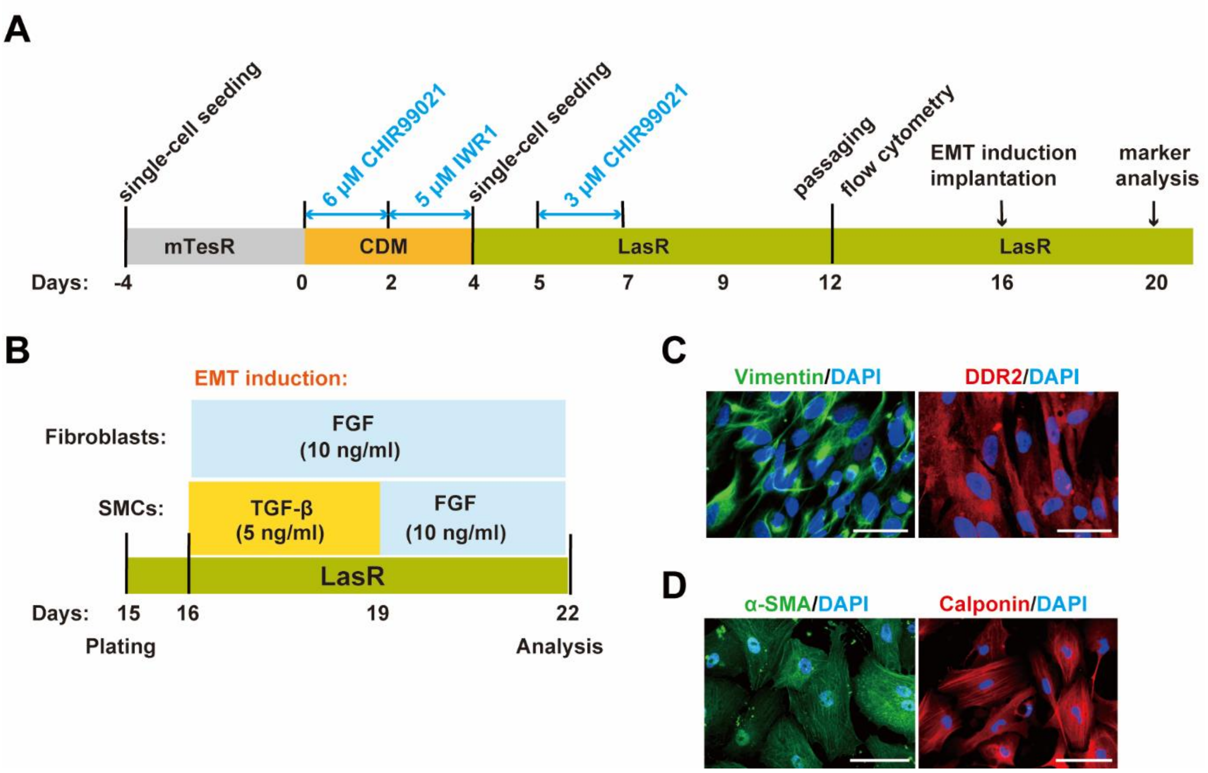

Figure S1. Schematic for differentiation of human embryonic stem cells to epicardial cells (hEPs) and EMT property of hEPs. A) Schematic for hEP differentiation. CDM, chemical defined medium (RPMI-1640 supplement with 213 μg mL^{-1} L-ascorbic acid 2-phosphate, and 2 mg mL^{-1} bovine serum albumin); LasR, advanced DMEM/F12 supplement with 1× GlutaMax, and 100 μg mL^{-1} L-ascorbic acid. B) Schematic for EMT induction from hEPs to fibroblasts and smooth muscle cells (SMCs). FGF, fibroblast growth factor; TGF-β, transforming growth factor β. C, D) Representative images of immunocytochemical staining for fibroblast markers (C) and SMC markers (D) following the EMT induction of hEPs. $n = 3$. Scale bar, 50 μm.

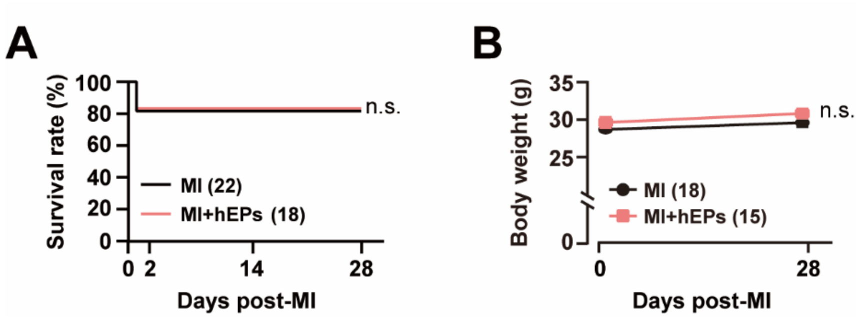

Figure S2. Survival rates and body weights of myocardial infarction (MI) mice with and without hEP treatment. A) Kaplan-Meier survival curves of mice. B) Body weights of the survived mice. Data are means ± S.E.M. Two-way ANOVA followed by Bonferroni's multiple analysis. n.s., no significant difference.

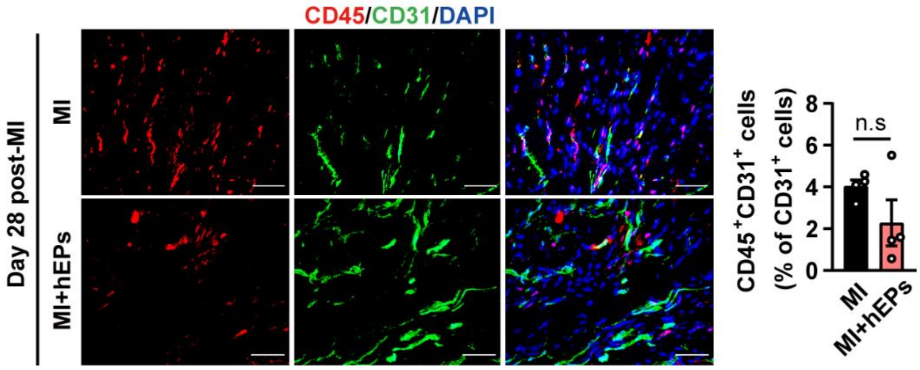

Figure S3. Representative images and quantitative data of immunofluorescent staining for the ratio of $CD45^{+}CD31^{+}$ cells to total $CD31^{+}$ cells in the border zones at day 28 post-MI. $n = 4$ hearts each. Scale bar, 50 μm. Data are means ± S.E.M. Unpaired student's *t*-test. n.s., no significant difference.

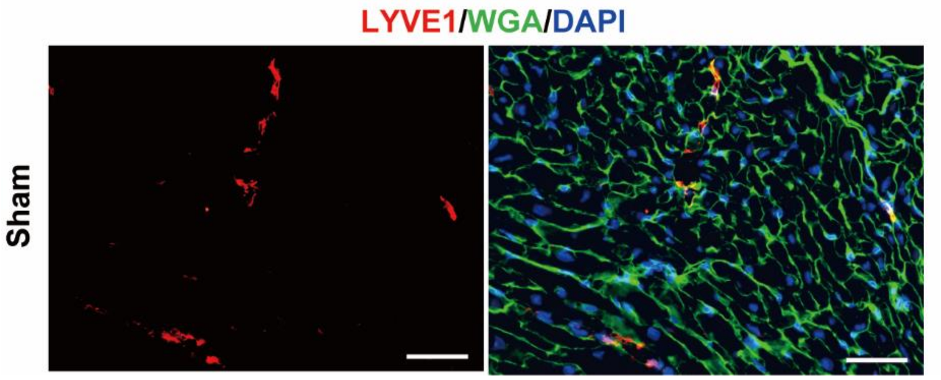

Figure S4. Representative images of immunofluorescent staining for lymphatic vessel endothelial hyaluronan receptor positive ($LYVE1^{+}$) lymphatic vessels. $n = 3$. Scale bar, 50 μm.

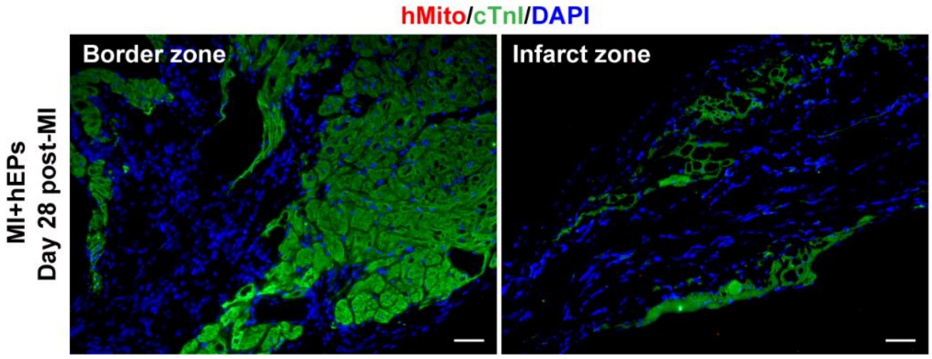

Figure S5. Immunofluorescent staining hardly detected human mitochondria positive (hMito$^+$) cells in the border and infarct zones of infarcted mouse hearts at day 28 post-MI. *n* = 6. For each heart, three sections from atrium to apex were analyzed. Whole visual fields were viewed, and three fields were captured for each section. Scale bar, 50 µm.

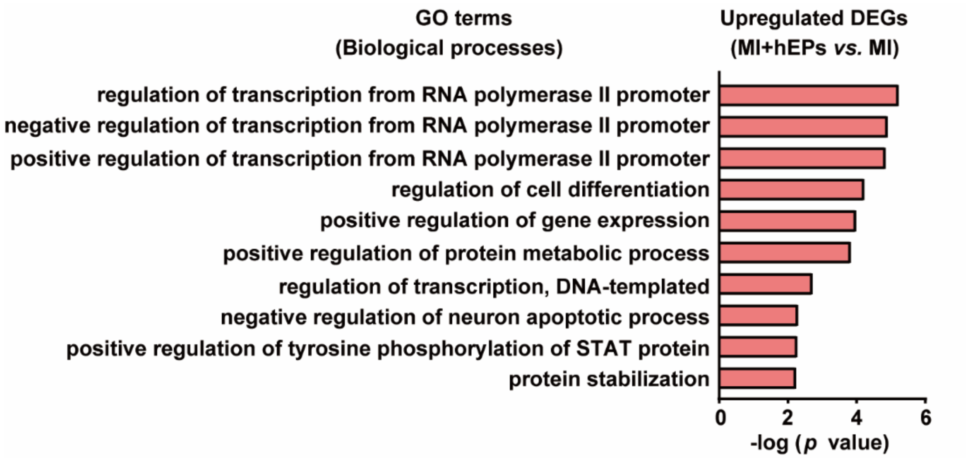

Figure S6. GO analysis of the biological processes in hEP-upregulated differentially expressed genes (DEGs). DEGs with p value < 0.05 and fold change ≥1.2 were analyzed.

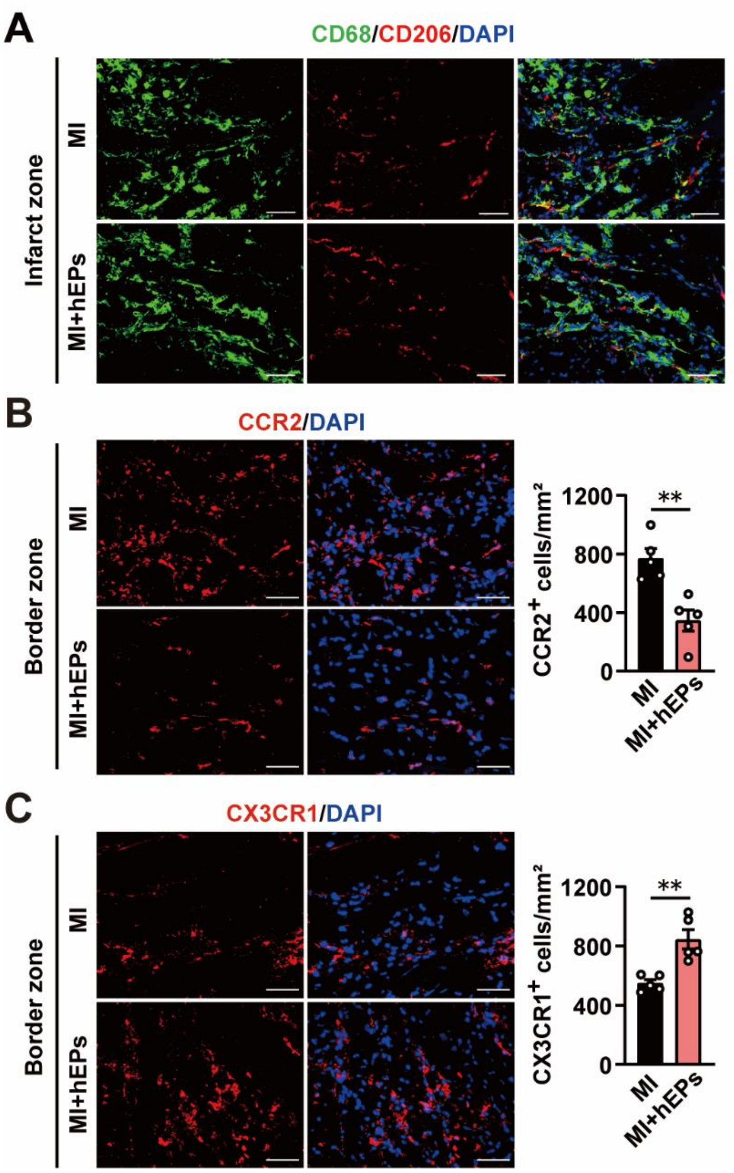

Figure S7. Macrophage infiltration and polarization in mouse infarcted hearts at day 3 post-MI. A) Representative images of immunofluorescent staining for $CD68^{+}$ cells and $CD68^{+}CD206^{+}$ macrophages. n = 6. Scale bar, 50 μm. Quantitative data was showed in Figure 3F. B, C) Representative images and quantitative data of immunofluorescent staining for the number of $CCR2^{+}$ cells (B) and $CX3CR1^{+}$ cells (C) in the border zones. n = 5 hearts each. Scale bar, 50 μm. Data are means ± S.E.M. Unpaired student's t-test. $^{**}p < 0.01$.

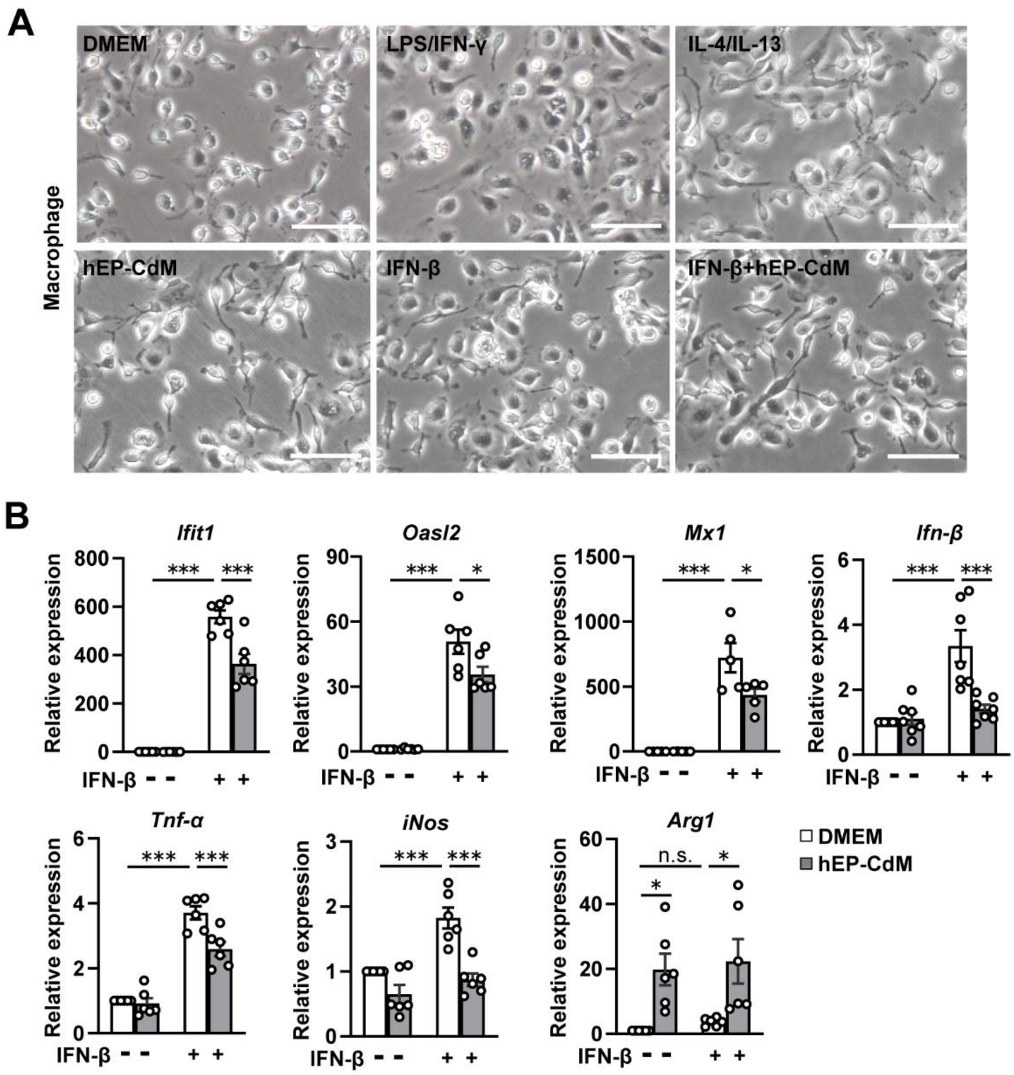

Figure S8. The hEP-conditioned medium (hEP-CdM) inhibits the transcript level of type I interferon-stimulated genes (ISGs) and pro-inflammatory genes while increases the reparative marker transcript in mouse peritoneal macrophages. A) Representative images showing the morphology of macrophages after being treated with serum-free DMEM vehicle control, LPS (50 ng mL^{-1})/IFN-γ (100 ng mL^{-1}), IL-4 (10 ng mL^{-1})/IL-13 (10 ng mL^{-1}), hEP-CdM, IFN-β (100 U mL^{-1}), or IFN-β + hEP-CdM for 24 h. n = 4. Scale bar, 50 μm. B) qPCR analysis of the transcript levels of ISGs and macrophage subtype markers with or without the 24 h-treatment of IFN-β (100 U mL^{-1}) and/or hEP-CdM. n = 5 to 7. Data are means ± S.E.M. Two-way ANOVA followed by Bonferroni's multiple analysis. $^{*}p < 0.05$, $^{***}p < 0.001$. n.s., no significant difference.

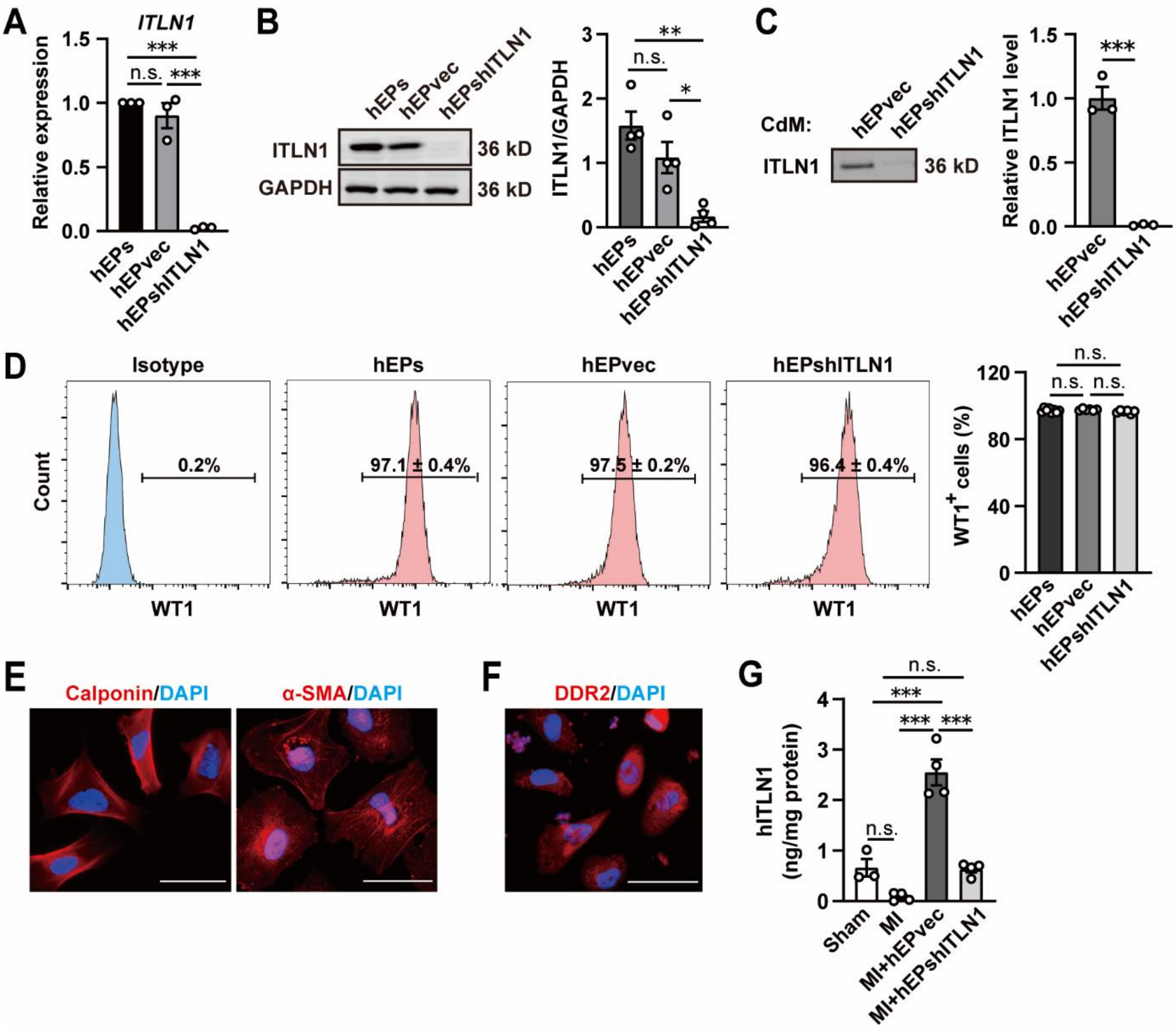

Figure S9. Effects of ITLN1 knockdown in hEPs. A) qRT-PCR analysis of the *ITLN1* level in hEPs at 5 days post-lentivirus transfection. *n* = 3. hEPvec, hEPs transfected with lentivirus packaged with the vector control; hEPshITLN1, hEPs transfected with lentivirus packaged with *ITLN1* shRNA. B, C) Western blotting analysis of the ITLN1 level in the hEPs (B, *n* = 4) and the CdM collected from hEPs (C, *n* = 3) at 5 days post-lentiviral transfection. D) Flow cytometry analysis and quantitative data of the percentage of $WT1^+$ hEPs at 5 days post-lentivirus transfection. *n* = 4. E, F) Immunocytochemical staining of the SMC markers (E, *n* = 3) and fibroblast marker (F, *n* = 3) at day 6 after EMT induction of the hEPshITLN1. Scale bar, 50 μm. G) ELISA analysis of the hITLN1 level in the infarct and border zones of LV tissues at day 1 post-MI. *n* = 3 to 4. Data are means ± S.E.M. One-way ANOVA followed by

Tukey's multiple comparison test in A, B, D, and G. Unpaired student's *t*-test in C. $^{*}p < 0.05$, $^{**}p < 0.01$, $^{***}p < 0.001$; n.s., no significant difference.

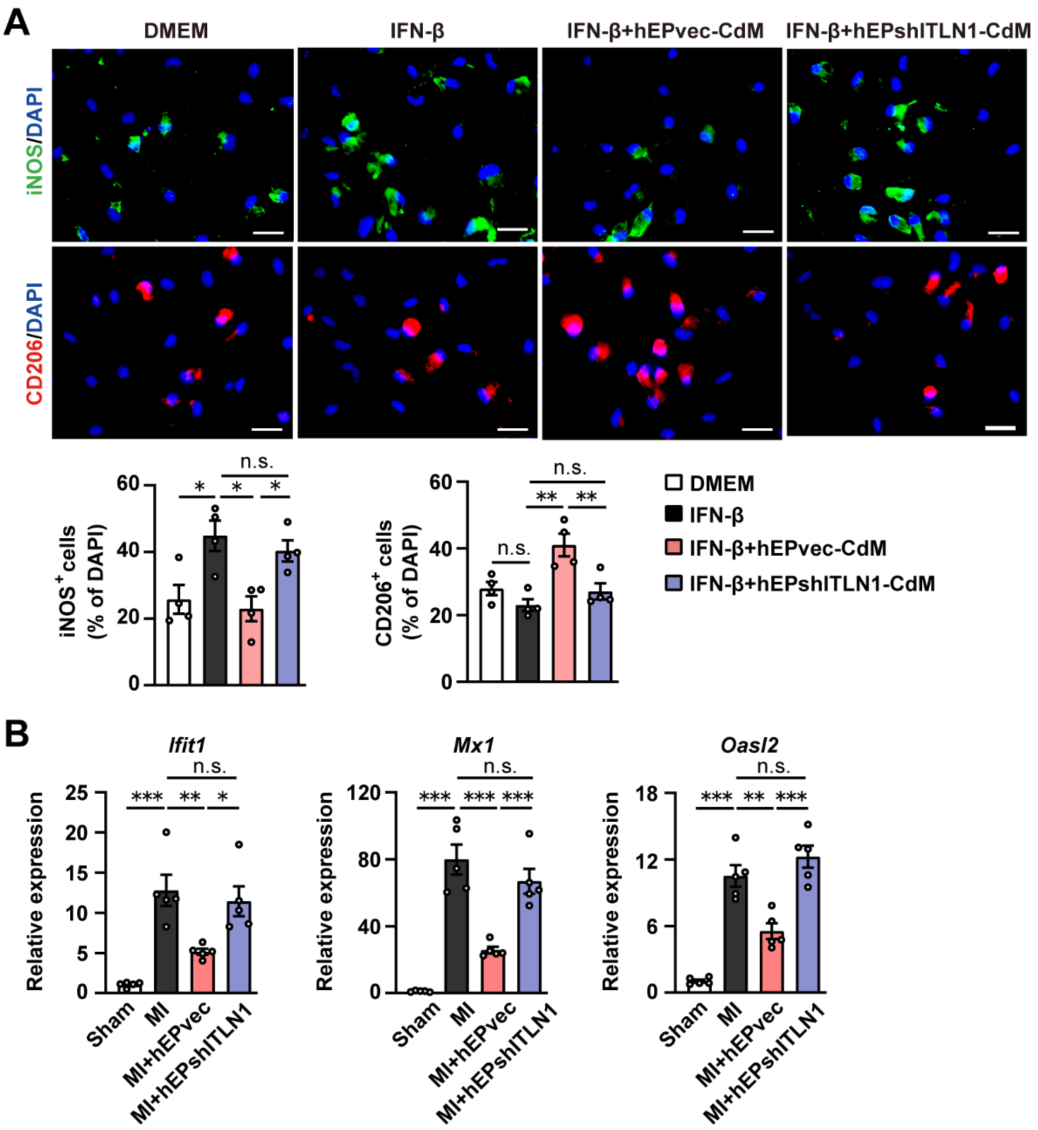

Figure S10. The effects of ITLN1 knockdown hEPs on macrophage polarization *in vitro* and IFN-I responses *in vivo*. A) Representative images and quantification of immunofluorescent staining for $iNOS^+$ and $CD206^+$ macrophages *in vitro*. $n = 4$. Scale bar, 20 μm. B) qRT-PCR analysis of the transcriptional levels of ISGs in the LV tissues at day 3 post-MI. $n = 5$ hearts each. Data are means ± S.E.M. One-way ANOVA followed by Tukey's multiple comparison test. $^{*}p < 0.05$, $^{**}p < 0.01$, $^{***}p < 0.001$. n.s., no significant difference.

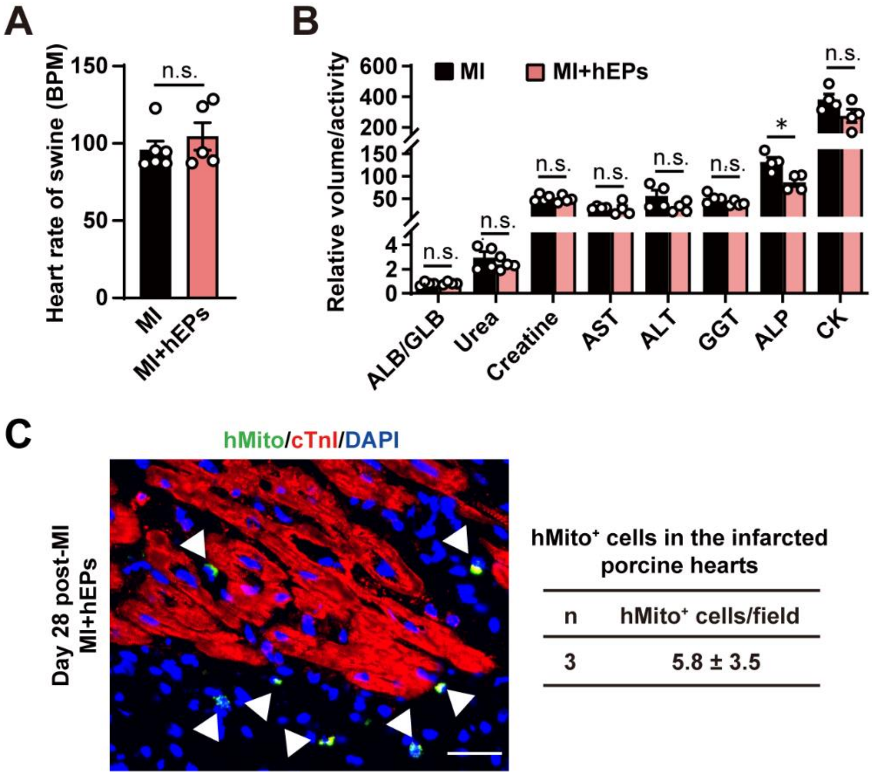

Figure S11. Heart rate measurements and serum chemistry analysis in swine at day 28 post-MI. A) Heart rate of swine was measured via LabChart. *n* = 6. B) Analysis of serum chemistry on renal (ALB/GLB ratio, urea, and creatine), hepatic (AST, ALT, GGT, and ALP), and cardiac (CK) indicators. *n* = 4. C) Representative image and quantitative data of $hMito^+$ cells in the infarcted porcine hearts. *n* = 3 hearts. Two to four fields from two sections of each heart were analyzed. Scale bar, 50 μm. Data are means ± S.E.M. Unpaired student's *t*-test. $^*p < 0.05$. n.s., no significant difference. ALB/GLB, albumin/globulin ratio; AST, aspartate aminotransferase; ALT, alanine aminotransferase; GGT, γ-glutamyl transferase, ALP, alkaline phosphatase; CK, creatine kinase.

Table S1. GO annotation of the hEP-downregulated genes in the infarcted left ventricles

ID	Term	Count	Genes	*P* value
GO:0002376	**Immune system process**	30	*Ifitm3, Cd5l, Pik3cd, Mefv, Ifi30, Ly9, Pycard, Adgre1, Inpp5d, Cd300lb, Slamf7, Cd300lf, Cd177, Eomes, Cd74, H2-eb1, Fcer1g, Icosl, H2-aa, Tlr1, Lat2, Bst2, Tyrobp, Themis2, Naip6, Btk, Sh2d1b2, Padi4, Myd88, H2-ab1*	1.93E-24
GO:0006954	**Inflammatory response**	16	*Ccl12, Slc11a1, Ptafr, Cd5l, Pik3cd, Cyba, Tcirg1, Mefv, Pycard, Tlr1, Cxcl10, Themis2, Naip6, Chil3, Myd88, Ccr2*	9.24E-11
GO:0045087	**Innate immune response**	19	*Ifitm3, Fcer1g, Mx1, Pik3cd, Cyba, Mefv, Ly9, Rnaset2a, Pycard, Tlr1, Bst2, Ifi27, Naip6, Btk, Sh2d1b2, Slamf7, Padi4, Myd88, Cd177*	8.61E-10
GO:0002250	**Adaptive immune response**	13	*Eomes, Cd74, H2-eb1, Pik3cd, Icosl, H2-aa, Ly9, Adgre1, Lat2, Btk, Sh2d1b2, Slamf7, H2-ab1*	1.81E-09
GO:0006955	**Immune response**	16	*Cd74, Ccl12, H2-eb1, H2-t10, H2-bl, Was, H2-aa, Vav1, Gm8909, Tlr1, Cxcl10, Pnp, Enpp2, Myd88, Ccr2, H2-ab1*	2.16E-09
GO:0034341	**Response to interferon-gamma**	7	*Ifitm3, Bst2, Cd74, H2-eb1, Slc11a1, Mefv, H2-aa*	4.88E-09
GO:0019886	**Antigen processing and presentation of exogenous peptide antigen via MHC class II**	6	*Cd74, H2-eb1, Fcer1g, Ifi30, H2-aa, H2-ab1*	1.76E-08
GO:0050870	**Positive regulation of T cell activation**	6	*Pycard, H2-eb1, H2-aa, H2-ab1, Ccr2, Sirpb1c*	4.05E-07
GO:0032755	**Positive regulation of interleukin-6 production**	8	*Pycard, Tlr1, Cd74, Tyrobp, Fcer1g, Ptafr, Cyba, Myd88*	5.18E-07
GO:0032760	**Positive regulation of tumor necrosis factor production**	8	*Pycard, Tlr1, Tyrobp, Fcer1g, Ptafr, Cyba, Myd88, Ccr2*	7.79E-07

Table S2. Primer sequences for qRT-PCR

Names	Primer sequences (5’-3’)
Il-6	F- GATGGATGCTACCAAACTGGAT<br>R- CCAGGTAGCTATGGTACTCCAGA
Ccl7	F- GCTGCTTTCAGCATCCAAGTG<br>R- CCAGGGACACCGACTACTG
Cxcl10	F- CCAAGTGCTGCCGTCATTTTC<br>R- GGCTCGCAGGGATGATTTCAA
Ifit1	F- CTGAGATGTCACTTCACATGGAA<br>R- GTGCATCCCCAATGGGTTCT
Mx1	F- GACCATAGGGGTCTTGACCAA<br>R- AGACTTGCTCTTTCTGAAAAGCC
Oasl2	F- TTGTGCGGAGGATCAGGTACT<br>R- TGATGGTGTCGCAGTCTTTGA
Ifn-β	F- TGGGTGGAATGAGACTATTGTTG<br>R- CTCCCACGTCAATCTTTCCTC
Tnf-α	F- GGGACAGTGACCTGGACTGT<br>R- CTCCCTTTGCAGAACTCAGG
iNos	F- ATGTCCGAAGCAAACATCAC<br>R- TAATGTCCAGGAAGTAGGTG
Gapdh	F- GTGGCAAAGTGGAGATTGTTG<br>R- CTCCTGGAAGATGGTGATGG
APOE	F- GTTGCTGGTCACATTCCTGG<br>R- GCAGGTAATCCCAAAAGCGAC
ITLN1	F- ACGTGCCCAATAAGTCCCC<br>R- CCGTTGTCAGTCCAACACTTTC
IGFBP2	F- GACAATGGCGATGACCACTCA<br>R- CAGCTCCTTCATACCCGACTT
ANXA2	F- GAGCGGGATGCTTTGAACATT<br>R- TAGGCGAAGGCAATATCCTGT
GAPDH	F- GGAGCGAGATCCCTCCAAAAT<br>R- GGCTGTTGTCATACTTCTCATGG
hITLN1 shRNA (TRCN0000 159401)	F- 5'-CCGGGAATGTCCTAGTGCATTTGATCTC GAGATCAAATGCACTAGGACATTCTTTTTG-3'<br>R- 5'-AATTCAAAAAGAATGTCCTAGTGCATTT GATCTCGAGATCAAATGCACTAGGACATTC-3'

[a]F, forward; R, reverse; [b]*Gapdh*, mouse primer; *GAPDH,* human primer.
